# Genome Duplication and Gene Loss Affect the Evolution of Heat Shock Transcription Factor Genes in Legumes

**DOI:** 10.1371/journal.pone.0102825

**Published:** 2014-07-21

**Authors:** Yongxiang Lin, Ying Cheng, Jing Jin, Xiaolei Jin, Haiyang Jiang, Hanwei Yan, Beijiu Cheng

**Affiliations:** 1 Key Lab of Crop Biology of Anhui Province, School of Life Sciences, Anhui Agricultural University, Hefei, Anhui, China; 2 Crop Research Institute, Anhui Academy of Agricultural Sciences, Hefei, Anhui, China; University of Lausanne, Switzerland

## Abstract

Whole-genome duplication events (polyploidy events) and gene loss events have played important roles in the evolution of legumes. Here we show that the vast majority of Hsf gene duplications resulted from whole genome duplication events rather than tandem duplication, and significant differences in gene retention exist between species. By searching for intraspecies gene colinearity (microsynteny) and dating the age distributions of duplicated genes, we found that genome duplications accounted for 42 of 46 Hsf-containing segments in *Glycine max*, while paired segments were rarely identified in *Lotus japonicas*, *Medicago truncatula* and *Cajanus cajan*. However, by comparing interspecies microsynteny, we determined that the great majority of Hsf-containing segments in *Lotus japonicas*, *Medicago truncatula* and *Cajanus cajan* show extensive conservation with the duplicated regions of *Glycine max*. These segments formed 17 groups of orthologous segments. These results suggest that these regions shared ancient genome duplication with Hsf genes in *Glycine max*, but more than half of the copies of these genes were lost. On the other hand, the *Glycine max* Hsf gene family retained approximately 75% and 84% of duplicated genes produced from the ancient genome duplication and recent *Glycine*-specific genome duplication, respectively. Continuous purifying selection has played a key role in the maintenance of Hsf genes in *Glycine max*. Expression analysis of the Hsf genes in *Lotus japonicus* revealed their putative involvement in multiple tissue-/developmental stages and responses to various abiotic stimuli. This study traces the evolution of Hsf genes in legume species and demonstrates that the rates of gene gain and loss are far from equilibrium in different species.

## Introduction

Whole-genome duplication, or polyploidy, is a common phenomenon in the evolution of plants and is particularly widespread in angiosperms [1], [2]. Many modern diploid plants have experienced one or more episodes of polyploidy and possess vestiges of multiple rounds of polyploidy [3], [4]. Recently, comparisons between legume genomes have revealed that legumes of the large Papilionoideae subfamily (papilionoids) have undergone whole-genome duplication [5]. This older shared polyploidy event is estimated to have occurred 56 to 65 million years ago (Mya) [6], [7]. A second, more recent genome duplication event occurred only in the lineage leading to *Glycine* up to 13 Mya [8]. Over time, the genomes of these plants diploidized, accompanied by rearrangements and loss of genes and chromosomal segments; this eliminated much of the evidence of the original duplication. Genome duplication and subsequent fractionation have played key roles in shaping present-day legume genomes [9].

A gene family is a group of similar genes resulting from wholesale or partial gene duplication; the size of a gene family reflects the number of duplicated genes (paralogs) in each species [10]. Whole-genome duplication causes each gene in the genome to be present in two copies. However, after duplication, not all categories of genes respond the same way to polyploidy; gene loss often occurs independently in different gene families. Some gene families, such as NBS-LRR resistance genes, show rapid rates of turnover among family members, with the loss of major gene lineages in some plant families [11], [12]. Alternatively, most genes are retained in other families with highly conserved amino acid sequences, such as transcription factors [13]. In the *Arabidopsis* genome, three whole-genome duplications that have occurred in the past 350 million years have brought about a greater than 90% increase in the number of transcription factor, signal transduction and developmental genes [14]. Genes retained after polyploidy may buffer critical functions, but over time, a gradual erosion of this capacity of duplicated genes may contribute to the cyclicality of genome duplication [15].

Here, we examine the evolution of the heat shock transcription factor (Hsf) family in legume species. Hsfs serve as the terminal components of signal transduction and are the central regulators of the expression of heat shock proteins and other heat shock-induced genes that confer thermotolerance to all eukaryotes [16]–[21]. Like many other transcription factors, Hsfs have a modular structure [22]. Hsf proteins share a well-conserved DNA binding domain (DBD) at their N termini, an adjacent bipartite oligomerization domain (HR-A/B region) composed of hydrophobic heptad repeats, a nuclear location signal (NLS), a nuclear export signal (NES) and a C-terminal activation domain (AHA motifs) [16], [23]–[26]. In contrast to the small number of Hsf genes found in *Drosophila*, *Caenorhabditis elegans*, yeasts and animals [27]–[33], the Hsf system is more complex in plants than in any other organisms investigated thus far. To date, on the basis of genome-wide analysis, 21 [34], approximately 25 [35], [36] and 25 [37] Hsf genes have been identified in the model plants *Arabidopsis*, rice and maize, respectively. On the basis of sequence divergence, three Hsf classes (A, B and C) and several subgroups are currently recognized [22]. Previous studies have shown that there are no apparent tandem duplications, and no clustered organization, in the Hsf families of several monocot and eudicot species [35], [37]. How did the members of this gene family arise, and how are the copy numbers of genes in this family maintained? Multiple rounds of genome duplication and extensive gene loss in different plant lineages may have led to the generation of independent growth and evolution models of Hsf family genes.

In this study, we analyzed the Hsf gene families from six papilionoid legume species for which substantial information about genomes or transcriptomes was available, namely *Lotus japonicus* (birdsfoot trefoil), *Medicago truncatula* (barrel medic), *Cicer arietinum* (chickpea), *Glycine max* (soybean), *Cajanus cajan* (pigeonpea) and *Phaseolus vulgaris* (common bean). The aim of this investigation was to determine which genes were derived from genome duplication, subsequently giving rise to paralogs, which genes descended from speciation events, giving rise to orthologs and which genes have undergone gene loss. In addition to examining the phylogeny of the Hsf family, we performed a comprehensive examination of the legume genome structure anchored by Hsf genes. Furthermore, we searched for gene microsynteny within and between the genomes of the legumes to investigate the evolutionary history of the Hsf regions. Our data show that extensive synteny remains in the homeologous regions within/between legume species. In addition, most Hsf regions can be traced to ancient papilionoid-specific whole-genome duplication or recent *Glycine*-specific whole-genome duplication. However, different rates of gene loss in the Hsf family have occurred along separate lineages of legume. Our results may help facilitate the extrapolation of Hsf gene function from one lineage to another.

## Materials and Methods

### Data retrieval and sequence analysis

The most recent versions of genome, protein and cDNA sequences of each species were downloaded from the respective genome sequence sites as follows: *L*. *japonicus* (version 2.5) from the *L. japonicus* Genome Sequencing Project (http://www.kazusa.or.jp/lotus/), *M*. *truncatula* (version 3.5) from the *M. truncatula* Genome Sequencing Project (http://www.medicagohapmap.org/?genome), *G. max* (version 1.01) from the Soybean Genome Sequencing Project (http://www.phytozome.net/soybean.php), *C. cajan* (version 1.0) from the International Crops Research Institute for the Semi-Arid Tropics (http://www.icrisat.org/gt-bt/iipg/Genome_Manuscript.html), *P. vulgaris* (version 0.9) from the US Department of Energy Joint Genome Institute (http://www.phytozome.net/commonbean.php), *P. patens* (version 1.6) from the Joint Genome Institute (http://www.phytozome.net/physcomitrella), *S. moellendorffii* (version 1.0 filtered model 3) from the Joint Genome Institute (http://genome.jgi-psf.org/Selmo1/Selmo1.download.ftp.html) and *C. reinhardtii* (version 4.3) from the Joint Genome Institute (http://www.phytozome.net/chlamy.php). Although information about the whole genome of *C. arietinum* is not currently available, the *C. arietinum* transcriptome has been sequenced using next-generation sequence technology [38] and was obtained from the Chickpea Transcriptome Database (http://www.nipgr.res.in/ctdb.html). The downloaded nucleotide and protein sequences of each species were in turn used to build local databases using DNATOOLS software. Published Hsf protein sequences [34], [35], [39] were used to search the Pfam database [40], and an integrated and exactly conserved Hsf-type DBD domain sequence based on the Hidden Markov Model (HMM) was obtained. The Hsf domain (PF00447) in the Pfam HMM library was then used in BLASTP searches to identify Hsfs from the local databases. For the *C. arietinum* transcriptome, a TBLASTN search was performed, and the identified full-length cDNAs were translated in the correct frame. Only hits returning E-values of less than 0.001 were considered for further analysis. This step was crucial for finding as many similar sequences as possible. Moreover, on the basis of BLASTN search results in the genome databases using the predicted cDNA sequences of Hsf genes, information was obtained about the chromosome locations of these genes. Redundant sequences with different identification numbers and the same chromosome locus were eliminated from the data set. To confirm the presence of both Hsf-type DBD domain and HR-A/B regions in the sequences obtained, the predicted protein sequences of Hsf genes were analyzed in the Pfam HMM database and the SMART tool [41] to find the DBD domain, and proteins without these regions were excluded from the data set. Following this step, the remaining sequences were examined for the HR-A/B regions using the MARCOIL program [42] and the SMART tool, both of which can recognize the coiled-coil structure representing the core of the HR-A/B region; proteins without HR-A/B regions were removed from the data set.

In addition to the DBD and HR-A/B domains, many Hsfs also contain an NLS and an NES domain, and most plant class A Hsfs contain one or several AHA motifs. To identify the NLS domain in the Hsfs, the program PredictNLS [43] (from the website) was used. In addition, the NetNES 1.1 server [44] was used to detect the NES domain in all of the Hsfs. Moreover, since the highly conserved amino acid sequence of AHA motifs has been elaborated previously, and detailed investigations of these motifs have been reported [25], [26], [34], the AHA motifs could be predicted based on sequence comparisons and their characteristics. Information about the AHA motifs was further verified by alignments with published Hsf sequences.

### Multiple sequence alignments and phylogenetic analysis

Multiple sequence alignments using ClustalX (version 1.83) [45] were performed on the N-terminal domains of the Hsfs obtained, including the DBD domains, the HR-A/B regions and parts of the linker between these regions. The alignment was then adjusted manually by Jalview. A phylogenetic tree was constructed with the aligned protein sequences using MEGA (version 4.0) [46] using the NJ method with the following parameters: Poisson correction, pairwise deletion and bootstrap (1,000 replicates; random seed). The Hsf of *Saccharomyces cerevisiae* (ScHsf1), and the Hsfs of *C. reinhardtii*, *S. moellendorffii* and *P. patens*, were used as the outgroup. In order to analyze the classes and subgroups of the legume Hsf families, 25 maize Hsfs (ZmHsfs) [37], 25 rice Hsfs (OsHsfs) [35] and 21 *Arabidopsis* Hsfs (AtHsfs) [34] were included in the phylogenetic analysis by generating a NJ tree (Poisson correction, pairwise deletion and bootstrap  =  1,000 replicates). To confirm the robustness of the NJ tree, we built the ML tree using maximum likelihood method (MEGA 6.0; bootstrap  =  1,000 replicates, amino acid substitution model, Jones-Taylor-Thornton matrix).

### Intraspecies microsynteny analysis

To categorize the expansion of the Hsf gene families, the physical locations of all members of this family were examined in *L. japonicus*, *M*. *truncatula*, *G. max* and *C. cajan*. Tandem duplication is characterized by multiple gene family members occurring within either the same or neighboring genomic regions. Tandem duplicated genes were defined as genes in any gene pair, T1 and T2, that (1) belong to the same gene family, (2) are located within 100 kb each other, and (3) are separated by zero, one or fewer, five or fewer, or 10 or fewer nonhomologous (not in the same gene family as T1 and T2) spacer genes [47]. A method similar to that of Maher *et al*. [48] and Zhang *et al*. [49] was implemented to identify large-scale duplication events. To classify two Hsf genes as residing within a duplicated block, their neighboring protein-coding genes must be highly similar at the amino acid level. First, all Hsf genes in each family were used as the original anchor points. Next, 15 protein-coding sequences upstream and downstream of each anchor point were compared by pairwise BLASTP analysis to identify duplicated genes between two independent regions. The software then counted the total number of protein-coding genes flanking any anchor point that had the best nonself match (*E-value* <10^−10^) with a protein-coding gene neighboring another anchor point. When four or more such gene pairs with syntenic relationships were detected, the two regions were considered to have originated from a large-scale duplication event.

### Interspecies microsynteny analysis

The analysis of microsynteny across species was based on comparisons of the specific regions containing Hsf genes. Similarly, the Hsf genes of *L. japonicus*, *M*. *truncatula*, *G. max* and *C. cajan* were set as the anchor points according to their physical locations. The protein-coding sequences assigned to the flanking regions of each Hsf gene in one species were aligned with those in the other species by pairwise comparisons. A syntenic block is defined as the region in which three or more conserved homologs (BLASTP *E-value* <10^−20^) were located within a 100 kb region between genomes [50].

### Duplication event dating and adaptive evolution analysis

The duplicated gene pairs within each duplicated block were used to calculate Ks and to analyze Ka/Ks ratios. Protein sequences of the gene pairs were aligned using MUSCLE [51], and the results were used to guide the codon alignments by PAL2NAL [52]. The generated codon alignments were subjected to computation of Ks and divergence levels (Ka/Ks ratios) using DnaSP software (version 5.10). A sliding window analysis of Ka/Ks ratios was performed with the following parameters: window size, 150 bp; step size, 9 bp.

When dating large-scale duplication events, Ks can be used as the proxy for time. For each pair of duplicated regions, the mean Ks of the flanking conserved genes were calculated, and these values were then translated into divergence time in millions of years assuming a rate of 6.1×10^−9^ substitutions per site per year. The divergence time (T) was calculated as T = Ks/(2×6.1×10^−9^) 10^−6^ Mya [53].

Codeml program is available under PAML (phylogenetic analysis maximum likelihood) V. 4.7 software [54]. To further assess whether positive selection acts upon specific sites, six site models that allow ω ratios (Ka/Ks ratios) to vary among sites, as implemented in the program Codeml, were used based on the coding sequences of Hsf genes [55]. These models are the one-ratio model (M0), the nearly neutral model (M1a), the positive-selection model (M2a), the discrete model (M3), the β model (M7) and the β & ω model (M8). The likelihood ratio tests (LRT) were performed to compare the corresponding models with and without selection (ie, M0 vs M3, M1a vs M2a, and M7 vs M8) [55]. M0–M3 comparison can be used to test whether ω values vary among sites. Both M1a–M2a and M7–M8 comparisons can be used to test positive selection acting on sites [55]. The Bayes empirical Bayes (BEB) were used in the M2a and M8 models to calculate the Bayesian posterior probability (BPP) of the codon sites under a positive selection [56].

### Microarray data

The data for evaluating Hsf gene expression in various tissues of *L. japonicus* acquired with the Lotus 52 K Affy chip were obtained from the Lotus Transcript Profiling Resource [57]. The locus names of Hsf genes in the *L. japonicus* Genome Sequencing Project were used to query the corresponding probe set IDs in the GeneChip. The log transformed expression values for the retrieved probe sets were then used to perform cluster analysis by Cluster [58].

### Plant material and stress treatments

*L. japonicus* plants (Miyakojima MG-20) were grown in a greenhouse at 25±2°C with a 14/10 h (light/dark) photoperiod. Four-week-old seedlings were prepared for abiotic stress treatments. For temperature treatments, the uniform-sized seedlings were transferred to the temperature-controlled growth chambers, which were maintained at 42±1°C for heat stress and at 4±1°C for cold stress. For oxidative stress, seedling leaves were sprayed with 10 mM H_2_O_2_ solution. After each treatment, the leaves of the seedlings were harvested at 0, 1 h and were immediately frozen in liquid nitrogen and kept at −80°C pending the extraction of RNA.

### Quantitative real-time PCR

Total RNA was extracted from the collected samples using Trizol reagent (Invitrogen, USA), followed by Dnase I digestion to remove residual genomic DNA contamination. The quality and quantity of the total RNA was measured by electrophoresis on 1% (w/v) agarose gels and examined with a NanoDrop ND-1000 UV-Vis spectrophotometer (NanoDrop Technologies, Inc.). For each sample, the first strand cDNA was reverse transcribed from 1 µg total RNA using the QuantiTect Rev. Transcription Kit (Qiagen, Germany) according to the manufacturer's instructions. Quantitative real-time PCR was conducted on an ABI PRISM 7300 real-time PCR system (Applied Biosystems, USA). All the gene-specific primer sequences for quantitative real-time PCR were designed by Primer Express Version 3.0 software (Applied Biosystems, USA) and are listed in Table S1. Each PCR reaction mixture contained 2.0 µL transcription product, 400 nM primers, and 12.5 µL 2×SYBR Green Master Mix Reagent (Applied Biosystems, USA) in a total volume of 25 µL. The thermal cycle used was as follows: 50°C for 2 min, 95°C for 10 min, 40 cycles of 95°C for 15 s, and 60°C for 1 min. Melting curve analysis was then used to verify the identity of the amplicons and the specificity of the reaction. To normalize the variance among samples, *β-tubulin* was used as an endogenous control. The relative expression of each gene was calculated as the ΔΔC_T_ value in comparison to unstressed samples (Applied Biosystems, USA). These experiments were independently replicated at least three times for each sample.

## Results

### Hsf genes form a complex family in legume genomes

Papilionoids represent all major legume crops and model legume species. Most papilionoid species fall into one of two large clades, i.e., the temperate galegoid clade (cool season legumes) and the Millettioid clade (tropical season legumes). To determine the number of full-length Hsf proteins in the six legumes, BLAST and HMM searches were performed against the annotated genomes of *L*. *japonicus*, *M*. *truncatula*, *G. max*, *C. cajan* and *P. vulgaris* as well as transcriptome data for *C. arietinum*. A total of 11, 19 and 13 Hsfs were identified in the cool season legumes *L*. *japonicus*, *M*. *truncatula* and *C. arietinum*, respectively, while 46, 22 and 29 Hsfs were identified in the tropical season legumes *G. max*, *C. cajan* and *P. vulgaris*, respectively (Table 1, Table S2). To obtain a broader perspective on the evolutionary history of the legume Hsf family, we also searched for Hsf genes in the single-celled green alga *Chlamydomonas reinhardtii*, the lycophyte *Selaginella moellendorffii* and the bryophyte *Physcomitrella patens*, which contain only two, one and seven Hsfs, respectively (Table 1, Table S2). Sequence alignment and domain analysis of the deduced Hsfs showed that the highly structured N-terminal DBD domain of each Hsf is the most conserved region, and the adjacent HR-A/B region, with a heptad pattern of hydrophobic amino acid residues, leads to the formation of a helical coiled-coil structure (Figure S1 and Figure S2; Table S3). These data indicate that the Hsf gene family has expanded in legumes relative to the basal plant taxa analyzed here, and to a greater extent in tropical season legumes than in cool season legumes.

**Table 1:** The number of Hsfs identified in the legume species and lower plants.

Class	LjHsf	MtHsf	CaHsf	GmHsf	CcHsf	PvHsf	SmHsf	PpHsf	CrHsf
A	9	12	8	26	14	17	0	3	1
B	2	7	4	19	7	11	1	4	1
C	0	0	1	1	1	1	0	0	0
Total	11	19	13	46	22	29	1	7	2

Lj  =  L. japonicus, Mt  =  M. truncatula, Ca  =  C. arietinum, Gm  =  G. max, Cc  =  C. cajan, Pv  =  P. vulgaris, Sm  =  S. moellendorffii, Pp  =  P. patens, Cr  =  C. reinhardtii.

**Figure 1:**
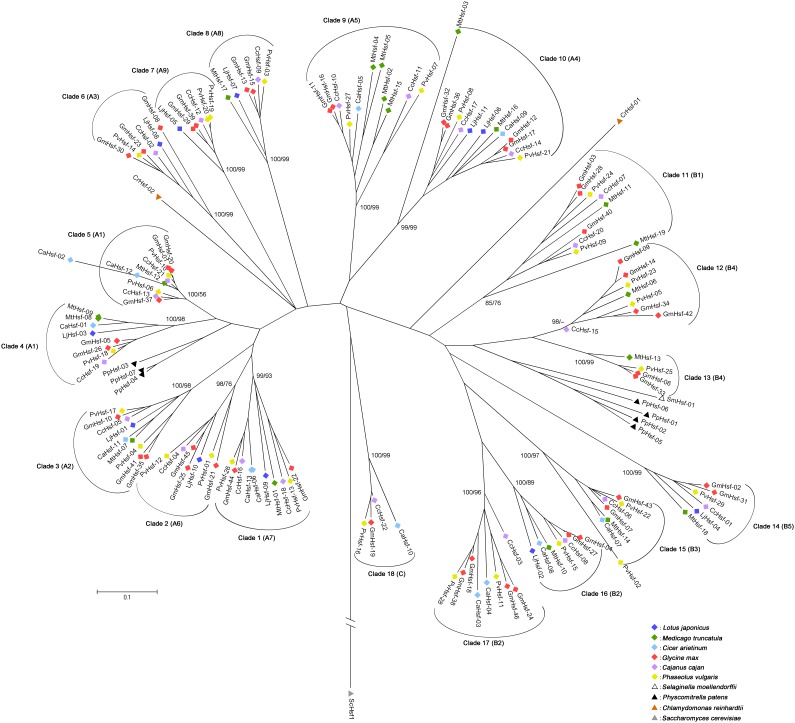
Phylogenetic tree of 140*L. japonicus*, *M. truncatula*, *C. arietinum*, *G. max*, *C. cajan* and *P. vulgaris*. This tree was constructed based on amino acid sequence comparison of the conserved N-terminal regions of Hsfs including the DNA-binding domain, the HR-A/B region and parts of the linker between them, using the neighbor-joining method with 1,000 bootstrap replicates. The Hsf of *Saccharomyces cerevisiae* (ScHsf1) and the Hsfs of *C. reinhardtii*, *S. moellendorffii* and *P. patens* were used as the outgroup. The colors indicate the species background of the Hsfs. The tree was divided into 18 shared clades (Clades 1–18) according to evolutionary distances. The bootstrap values of both neighbor-joining (NJ) tree (first number; 1000 replicates) and maximum likelihood (ML) tree (second number; 1000 replicates) were shown on the branches leading to each of the clades. The clades were supported by high bootstrap values in neighbor-joining and maximum likelihood analyses. Different subclasses of Hsfs are indicated in brackets. Gene names are presented in Table S2. The scale bar represents 0.1 amino acid changes per site.

Combining the six legume protein sequences, we constructed a phylogenetic tree using neighbor-joining analysis (Figure 1). The legume Hsfs were grouped into three major classes, A, B and C. Class A and B were further divided into nine (A1–9) and five (B1–5) subgroups with well-supported bootstrap values (Figure S3). Accordingly, previously defined classes and subgroups [22], [34] were identified from legume Hsfs (Figure S3).

Furthermore, phylogenetic analysis showed that the Hsf genes from the six legume species could be delineated into 18 well-supported ancient gene lineages (clades 1–18 in Figure 1), and strong amino acid sequence conservation was proven from the short branch lengths at the tips of the clades, indicating close evolutionary relationships among members. In each clade, branches with more than one Hsf gene from the same species are likely to have undergone gene duplication events, whereas the absence of representatives in some species is probably attributable to gene losses. In most cases, Hsf genes from tropical season legumes were more abundant than Hsf genes from cool season legumes. It is worth noting that in almost every clade, at least one extra copy of the Hsfs from *G. max* was present compared with that from the other species. On the other hand, in each clade, the members of different species may have evolved from a common ancestral gene by divergence of the lineage. Therefore, the 18 defined clades provide a framework for inferring parologs and orthologs of Hsfs. The phylogenetic relationships based on the ML tree were largely consistent with these results (Figure S4).

### Genome duplication played an important role in the expansion of the Hsf family

**Figure 2:**
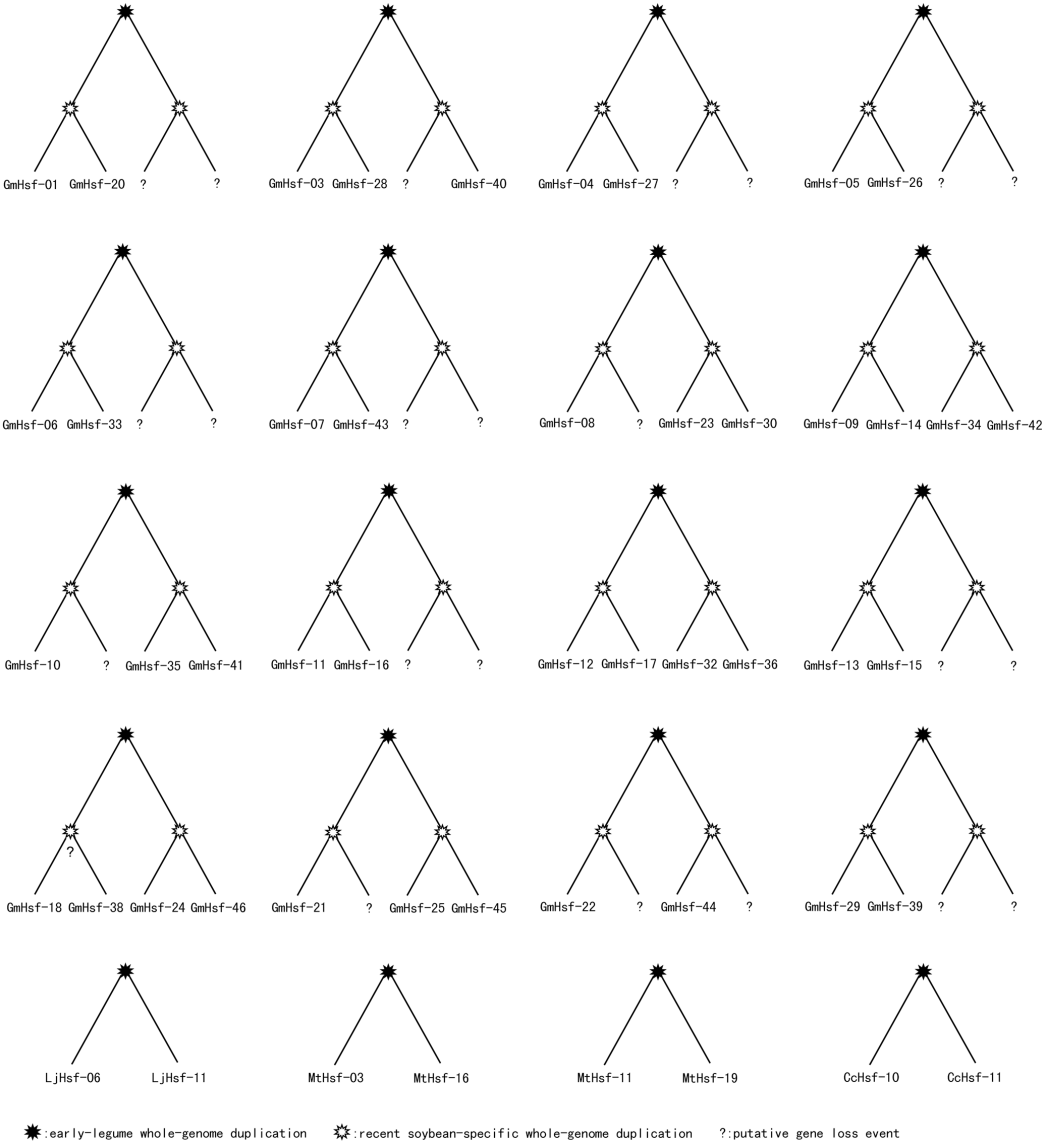
Idealized gene trees of the duplication groups of Hsf genes in *G. max*, *L*. *japonicus*, *M*. *truncatula* and *C*. *cajan*. Each tree represents a duplication group from large-scale gene duplication. As shown in the trees, every Hsf gene of *G. max* was expected to be present in four copies after two rounds of whole-genome duplication (early and recent). Similarly, the number of Hsf genes in *L*. *japonicus*, *M*. *truncatula* and *C*. *cajan* will have doubled after the early-legume whole-genome duplication. The five duplicated gene pairs (*LjHsf-06*/*LjHsf-11*, *GmHsf-09*/*GmHsf-34*, *GmHsf-18*/*GmHsf-24*, *GmHsf-18*/*GmHsf-46* and *GmHsf-21*/*GmHsf-45*) were classified in the flexible set. The question marks indicate possible gene loss events. The *GmHsf-18/GmHsf-38* pair could be formed by a segmental duplication that predated the recent whole-genome duplication, and both the *GmHsf-18* and *GmHsf-38* lost homoeologs from the recent whole-genome duplication.

To examine the relationship between the genetic divergences within each legume Hsf family and the corresponding expansion patterns, we further surveyed gene duplication events in the legume Hsf families (Figure 2; Table 2). *P. vulgaris* and *C. arietinum* were excluded from this analysis due to the lack of information about the locations of their Hsf genes. We characterized Hsf paralogs as being cluster or scattered. Chromosomal location analyses showed that the majority of Hsf genes are randomly scattered in the genomes, with tandemly clustered genes occurring in several places (Table S2). Legumes have experienced one or more polyploidy events. Thus, large-scale duplication events may have played an important role in the evolution of the legume Hsf families.

**Table 2:** Estimates of the dates for the large scale duplication events in legume species.

Duplicated Hsf gene pairs	Number of conserved flanking protein-coding genes	Ks (mean ± s.d.)	Date (mya)
*GmHsf-05* & *GmHsf-26*	14	0.12±0.02	9.84
*GmHsf-25* & *GmHsf-45*	15	0.12±0.01	9.84
*GmHsf-06* & *GmHsf-33*	16	0.13±0.01	10.66
*GmHsf-13* & *GmHsf-15*	16	0.13±0.02	10.66
*GmHsf-04* & *GmHsf-27*	14	0.14±0.02	11.48
*GmHsf-01* & *GmHsf-20*	16	0.15±0.01	12.30
*GmHsf-11* & *GmHsf-16*	15	0.15±0.02	12.30
*GmHsf-24* & *GmHsf-46*	16	0.15±0.02	12.30
*GmHsf-34* & *GmHsf-42*	12	0.16±0.02	13.11
*GmHsf-32* & *GmHsf-36*	11	0.16±0.01	13.11
*GmHsf-12* & *GmHsf-17*	14	0.16±0.01	13.11
*GmHsf-23* & *GmHsf-30*	7	0.17±0.02	13.93
*GmHsf-29* & *GmHsf-39*	16	0.17±0.03	13.93
*GmHsf-03* & *GmHsf-28*	16	0.18±0.02	14.75
*GmHsf-07* & *GmHsf-43*	6	0.18±0.03	14.75
*GmHsf-09* & *GmHsf-14*	16	0.18±0.03	14.75
*GmHsf-35* & *GmHsf-41*	7	0.18±0.06	14.75
*GmHsf-18* & *GmHsf-38*	5	0.38±0.10	31.15
*GmHsf-08* & *GmHsf-23*	4	0.57±0.02	46.72
*GmHsf-10* & *GmHsf-41*	7	0.58±0.11	47.54
*GmHsf-10* & *GmHsf-35*	6	0.59±0.10	48.36
*GmHsf-09* & *GmHsf-42*	4	0.62±0.04	50.82
*GmHsf-21* & *GmHsf-25*	5	0.62±0.08	50.82
*GmHsf-08* & *GmHsf-30*	4	0.65±0.07	53.28
*GmHsf-14* & *GmHsf-34*	4	0.65±0.06	53.28
*GmHsf-22* & *GmHsf-44*	8	0.65±0.07	53.28
*GmHsf-14* & *GmHsf-42*	5	0.66±0.04	54.10
*GmHsf-24* & *GmHsf-38*	5	0.71±0.10	58.20
*GmHsf-38* & *GmHsf-46*	6	0.72±0.10	59.02
*GmHsf-12* & *GmHsf-32*	6	0.77±0.04	63.11
*GmHsf-17* & *GmHsf-32*	6	0.77±0.06	63.11
*GmHsf-12* & *GmHsf-36*	6	0.79±0.04	64.75
*GmHsf-17* & *GmHsf-36*	6	0.80±0.06	65.57
*GmHsf-28* & *GmHsf-40*	4	0.85±0.17	69.67
*GmHsf-03* & *GmHsf-40*	4	0.91±0.12	74.59
*MtHsf-11* & *MtHsf-19*	5	0.77±0.10	63.05
*MtHsf-03* & *MtHsf-16*	5	0.80±0.03	65.66
*CcHsf-10* & *CcHsf-11*	5	0.74±0.14	60.66

The Hsf gene pairs from flexible sets were not used for calculation.

To investigate this possibility, we searched for gene similarity in the Hsfs flanking regions. If four or more of the 15 up- and downstream genes flanking two Hsf genes achieved a best non-self match using BLASTP (*E-value* <10^−10^), we considered these gene pairs to be conserved and defined these two regions as derived from a large-scale duplication event. We also defined a flexible set as a set of genes in which the flanking regions of an Hsf pair contained two or three conserved genes to avoid the possibility that pairs of Hsf genes resided within more divergent blocks.

We identified three conserved genes flanking the pair *LjHsf-06*/*LjHsf-11* in *L. japonicus*. Therefore, this pair is considered to have evolved from large-scale duplication, based on our flexible set. In *C. cajan*, one gene pair (*CcHsf-10*/*CcHsf-11*) was found to have involved large-scale duplication. However, it should be noted that approximately 45% of the Hsf family could not be assigned to any chromosome. In *M. truncatula*, genes flanking both pairs, *MtHsf-03*/*MtHsf-16* and *MtHsf-11*/*MtHsf-19*, were found to be conserved. In addition, two gene pairs (*MtHsf-04*/*MtHsf-05* and *MtHsf-08*/*MtHsf-09*) were located near each other on chromosomes 2 and 4, and thus most likely resulted from tandem duplication. Moreover, we found that the DNA sequences for *MtHsf-08* and *MtHsf-09*, as well as their four flanking genes (within an approximately 40-kb region) were completely identical to each other. In *G. max*, no tandem duplication was identified, but 42 out of 46 Hsf genes (approximately 91.3%) were arranged into duplicated chromosomal regions. These 42 genes were classified into 16 duplication groups; each group had two to four members that had conservation between their flanking genes (Figure 2). In the duplication groups of *G. max* Hsfs, the relationships between four putative duplicated gene pairs (*GmHsf-09*/*GmHsf-34*, *GmHsf-18*/*GmHsf-24*, *GmHsf-18*/*GmHsf-46* and *GmHsf-21*/*GmHsf-45*) were judged according to the flexible set.

**Figure 3:**
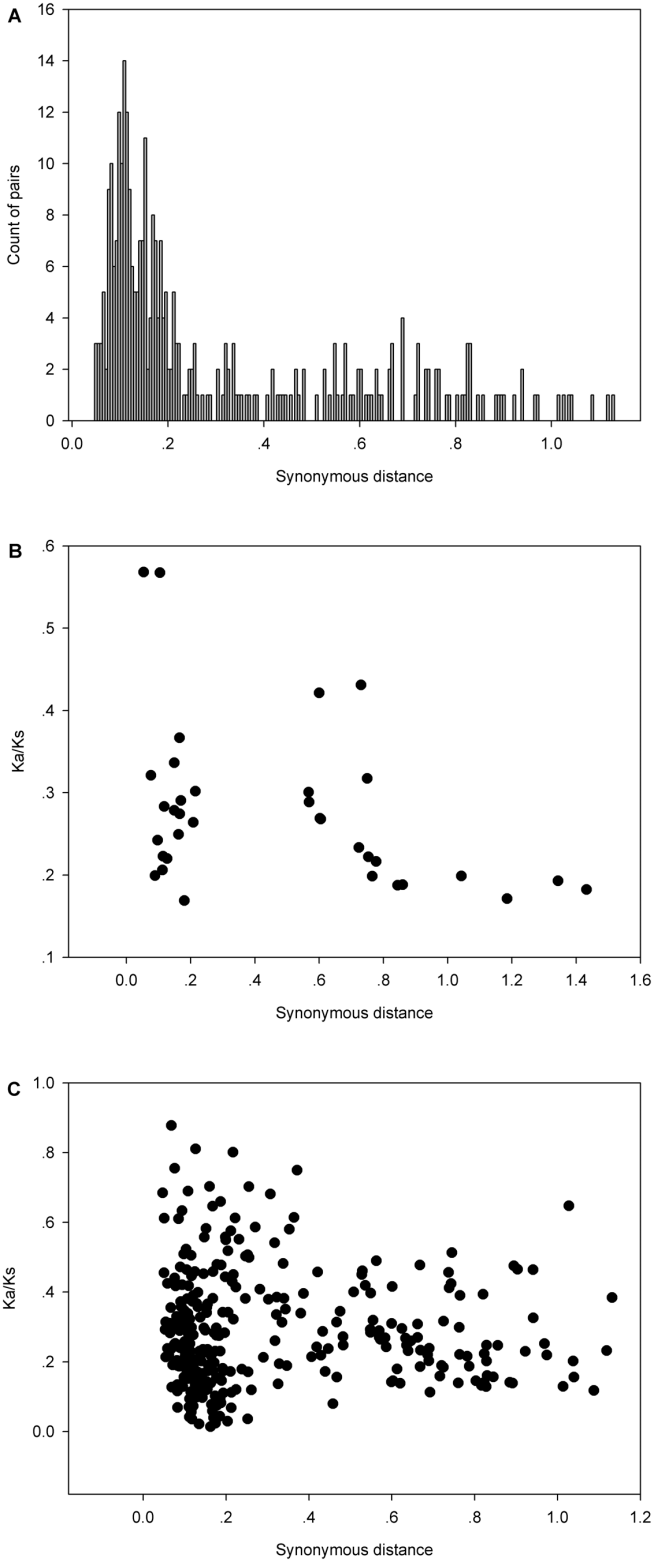
Estimates of Ks and Ka/Ks ratios in pairwise comparisons. (A) Distribution of synonymous distances (Ks) between paralogous genes flanking duplicated Hsf genes in *G. max*. The histogram shows the number of duplicate gene pairs (y-axis) versus synonymous distance between pairs (x-axis). The Ka/Ks ratios of the duplicated Hsf genes (B) and their flanking paralogs (C) in *G. max* are shown in the scatter plots; the y and x axes denote the Ka/Ks ratio and synonymous distance for each pair, respectively.

Assuming that synonymous silent substitutions per site (Ks) occur at a constant rate over time, the conserved flanking protein-coding genes were used to estimate the dates of the large-scale duplication events [48]. In this analysis, the duplicated blocks (excluding the flexible set) were used to date duplication events. The mean Ks values for each duplication event, and the estimated date, are shown in Table 2. The duplicated regions of *G. max* were divided into two groups (except for *GmHsf-18* and *GmHsf-38*) based on the Ks values of paralogs flanking the Hsf pair (Table 2). The Ks values distribution of each pair of genes in duplicated blocks is shown in Figure 3A. In one group, the paralogs flanking 17 Hsf pairs yielded a mean Ks value of 0.155 (the first peak in Figure 3A), corresponding to an event approximately 13 Mya. This estimate is consistent with the timing of a recent *Glycine*-lineage-specific tetraploidization event [8]. In the other group, the paralogs flanking 17 pairs had a mean Ks value of 0.701 (the second peak in Figure 3A), corresponding to an event roughly 57 Mya, concordant with the early-legume duplication that occurred near the origin of the papilionoid lineage [59]. For *GmHsf-18* and *GmHsf-38*, the duplication event was estimated to have occurred approximately 31 Mya, which is between the two rounds of genome duplication. In addition, *GmHsf-38* and *GmHsf-24* or *GmHsf-46* are all related via the ancient genome duplication, but the relationships between *GmHsf-18* and *GmHsf-24* or *GmHsf-46* are uncertain. Therefore, *GmHsf-18* may be the product of a segmental duplication of *GmHsf-38*. From these results, we conclude that two whole-genome duplications played a key role in the expansion of the *G. max* Hsf family.

In the *G. max* Hsf duplicated network, when two duplicated genes from recent duplication could not be found simultaneously, we reasoned that a possible ancient gene loss event occurred. As shown in Figure 2, in the ancestor of *G. max* lineage, ancient genome duplication should have produced at least 32 Hsf genes. However, eight of these lines lacked both copies, which would have been obtained from recent genome duplication, suggesting that approximately 25% of the ancient duplicates were lost over millions of years. Moreover, taking ancient gene losses into account, the number of *G. max* Hsf genes derived from recent genome duplication should be 50. Among these, eight pairs lost one copy of the gene, indicating that only about 16% of recent duplicates were lost. On the contrary, in *L. japonicas*, *M. truncatula* and *C. cajan*, only a few Hsf-containing segments could be matched in duplicated pairs. What is the origin of the remaining Hsf genes in these species?

### Massive losses of duplicated Hsf genes in *L. japonicas* and *M. truncatula*

**Figure 4:**
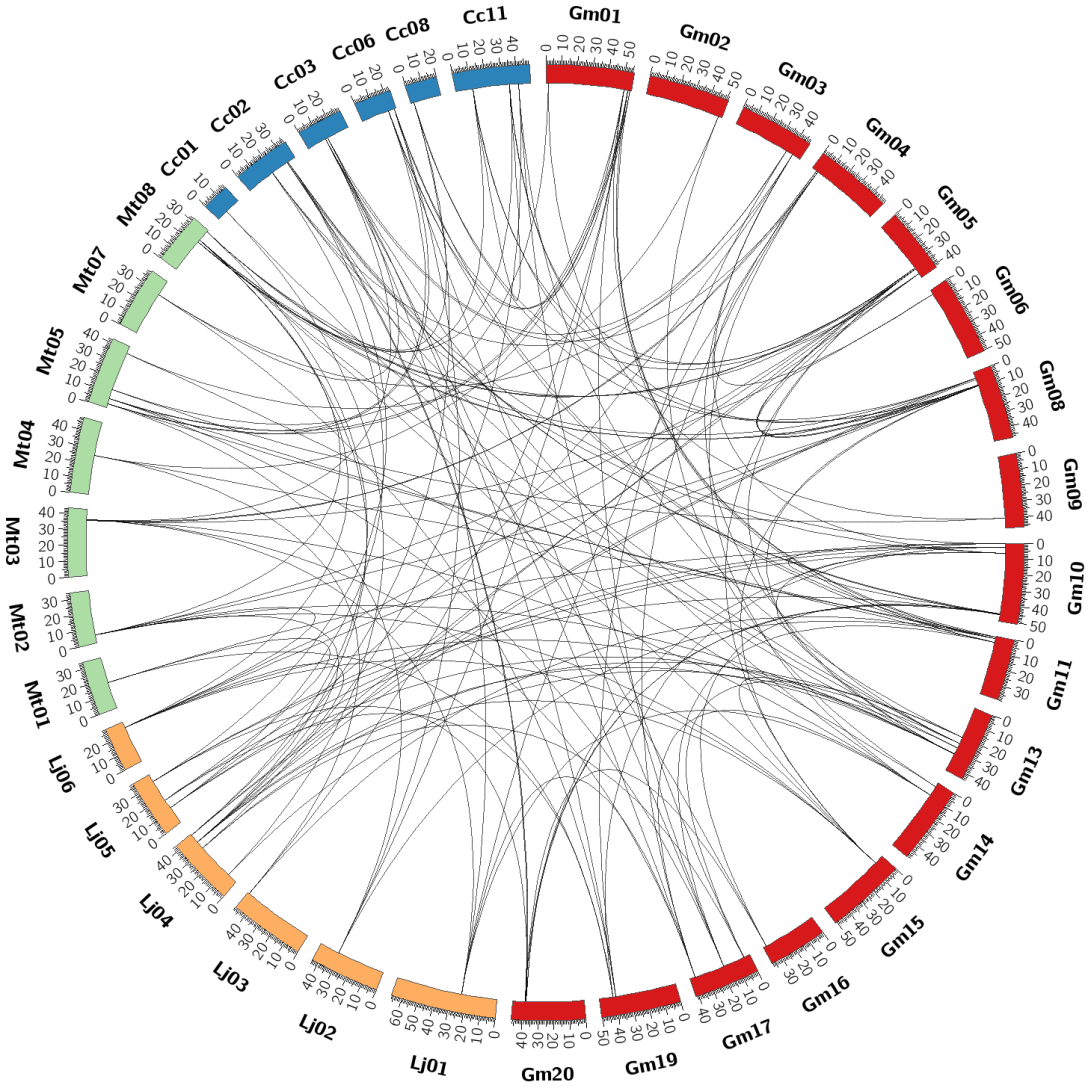
Extensive microsynteny of Hsf regions across *L*. *japonicus*, *M*. *truncatula*, *G. max* and *C. cajan*. *G. max* chromosomes, labeled Gm, are indicated by red boxes. The *L*. *japonicus*, *M*. *truncatula* and *C. cajan* chromosomes, shown in different colors, are labeled Lj, Mt and Cc, respectively. Numbers along each chromosome box indicate sequence lengths in megabases. The whole chromosomes of these four legumes, harboring Hsf regions, are shown in a circle. Black lines represent the syntenic relationships between Hsf regions.

**Figure 5:**
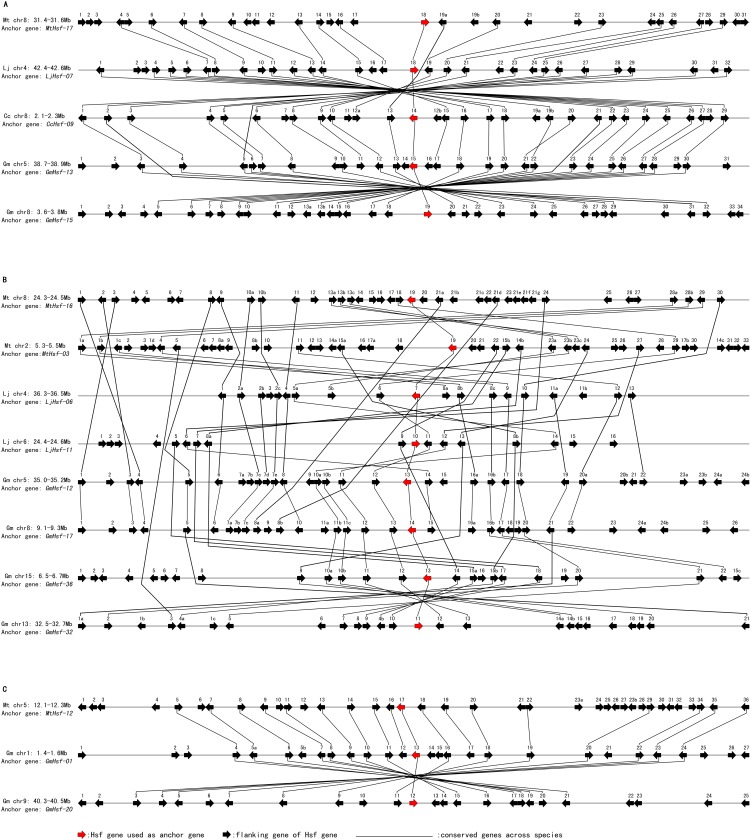
Comparative maps of representative Hsf genes and their flanking genes within syntenic chromosomal intervals across selected legume species. The relative positions of all flanking protein-coding genes were defined by the anchored Hsf genes, highlighted in red. The chromosome segments are shown as gray horizontal lines, with arrows corresponding to individual genes and their transcriptional orientations. All genes are numbered from left to right, in order, for each segment. Where several duplicated genes were present within a region, these genes were given the same number, with the letters a, b, c… appended in order. Conserved gene pairs among the segments are connected with lines. (A) The syntenic chromosomal intervals containing *MtHsf-17*, *LjHsf-07*, *CcHsf-09*, *GmHsf-13* and *GmHsf-15* across *M. truncatula*, *L. japonicus*, *C. cajan* and *G. max*. (B) The syntenic chromosomal intervals containing *MtHsf-03, MtHsf-16*, *LjHsf-06*, *LjHsf-11*, *GmHsf-12*, *GmHsf-17*, *GmHsf-32* and *GmHsf-36* across *M. truncatula*, *L. japonicus*, and *G. max*. (C) The syntenic chromosomal intervals containing *MtHsf-12*, *GmHsf-01* and *GmHsf-20* across *M. truncatula* and *G. max*. The full microsynteny maps of the regions containing Hsf genes within *M. truncatula*, *L. japonicus*, *C. cajan* and *G. max* are shown in Figure S5.

To identify the evolutionary origins and orthologous relationships within the Hsf genes of legumes, Hsf family members were used as anchor genes to study the molecular history of the chromosomal regions in which they reside. Using a stepwise gene-by-gene reciprocal comparison of the regions hosting the Hsf genes, we observed strongly conserved microsynteny among these regions across *L*. *japonicus*, *M*. *truncatula*, *G. max* and *C. cajan* (Figure 4 and Figure S5). After this interspecies microsynteny analysis, we were able to assemble 78 out of 86 Hsf-containing genomic segments from these four species into 17 groups (Figure S5 A–Q). We propose that all of the segments within a group descended from a single Hsf-containing segment in the genome of the last common ancestor of the legumes, and thus, we refer to these groups as orthologous groups. All of the groups contain at least one cool season legume and one tropical season legume segment with an Hsf gene. A total of 79 Hsfs (11 from *L*. *japonicus*, 16 from *M*. *truncatula*, 40 from *G. max* and 12 from *C. cajan*) were present in the 17 orthologous groups of segments. A representative synteny diagram for three of these groups is shown in Figure 5. *L*. *japonicas*-*M*. *truncatula*-*G. max*-*C. cajan* microsynteny also allowed us to verify the 18 ancient gene lineages inferred from the phylogenetic analysis. These results demonstrate that there is a one-to-one correspondence between syntenic orthologous groups and ancient gene lineages, except for the ancient gene of clade 18 (class C Hsfs).

Each orthologous group of segments includes between four and 24 orthologous groups of genes (average of 11; including Hsfs) with representation in at least two species. These groups are shown connected by black lines in Figure 5 and Figure S5; most of these groups include genes that obtain ‘best hits’ in BLASTP searches of entire genomes across species. To estimate the extent of conserved gene content and order, synteny quality was counted for those genes falling into syntenic intervals in *L*. *japonicus*, *M*. *truncatula*, *G. max* and *C. cajan*. Synteny quality was calculated as twice the number of matches divided by the total number of genes in both segments; this process discounts gene amplification but counts conservation of genes between species [5]. The average synteny quality of regions orthologous across these four species was 61.32% (Table 3). The lowest synteny quality, 48.97%, was between *M*. *truncatula* and *G. max* syntenic regions. The *G. max* and *C. cajan* comparison exhibited the highest conservation, 70.91%. These results support the orthology of the segment groups used in this study.

**Table 3:** The synteny quality of regions orthologous across *L. japonicus*, *M. truncatula*, *G. max* and *C. cajan.*

	*L. japonicus*	*M. truncatula*	*G. max*	*C. cajan*
*L. japonicus*				
*M. truncatula*	58.06%			
*G. max*	68.00%	48.97%		
*C. cajan*	70.79%	51.16%	70.91%	

In each orthologous group, high levels of microsynteny were maintained between the members of three legume species (*L. japonicus*, *M. truncatula* and *C. cajan*) and networks of duplicated regions in *G. max*, each anchored by the Hsf gene. Within an orthologous group, segments of different legume species are thought to have shared the ancient legume whole-genome duplication that occurred outside of the papilionoid lineage. In many groups, only one region of *L. japonicus*, *M. truncatula* and *C. cajan* was comparable to homoeologous regions of *G. max*, suggesting that one member of the Hsf gene pair produced from ancient genome duplication was lost in their ancestral lineages. For example, the *MtHsf-17*/*LjHsf-07*/*CcHsf-09* anchored regions showed microsynteny with two *G. max* duplicated regions containing *GmHsf-13/GmHsf-15* (Figure 5A). In only a few groups, two duplicated regions of *L. japonicus*, *M. truncatula* or *C. cajan* were syntenic to the *G. max* duplicate regions. In one instance (Figure 5B), where the *LjHsf-06*/*LjHsf-11* anchored regions were putative duplicated regions in *L. japonicus*, and the *MtHsf-03*/*MtHsf-16* anchored regions were duplicated regions in *M. truncatula*, these four regions could be aligned with four duplicated regions in *G. max* that contained syntenic counterparts of Hsf genes (*GmHsf-12*/*GmHsf-17/GmHsf-32/GmHsf-36*). The four *G. max* segments arose from two rounds of whole-genome duplication. Moreover, the Hsf orthologs were usually found in the syntenic regions of the three legume species, and there were no counterparts in one species of *L. japonicus*, *M. truncatula* or *C. cajan*, indicating that two Hsf copies produced from ancient genome duplication were lost in its ancestral lineage. We also uncovered four cases in which the regions containing Hsf orthologs was syntenic between only two legume species. *MtHsf-12* were conserved with those of *G. max* (*GmHsf-01*/*GmHsf-20*), while the orthologs of *MtHsf-12*/*GmHsf-01*/*GmHsf-20* were missing in *L*. *japonicus* and *C. cajan* (Figure 5C).

Because the 17 orthologous groups of Hsf-containing segments indicate that there are at least 17 Hsf genes in this ancestor, after ancient whole-genome duplication, 34 Hsf genes should have been produced in their progenitor. In nine orthologous groups, only one Hsf-containing region of *L. japonicus* showed microsynteny with the regions of other legumes, and in seven groups, the syntenic intervals anchored by Hsf genes were missing in *L. japonicus*. This indicates that 23 out of 34 ancient duplicated genes (approximately 68%) were lost in the *L. japonicas* Hsf family. Moreover, there were 11 orthologous groups with the single orthologous region in *M. truncatula* and four groups without sharing microsynteny with Hsf anchored regions in *M. truncatula*. This suggests that 19 out of 34 ancient duplicated genes (approximately 56%) were lost in the *M. truncatula* Hsf family. In *C. cajan*, all 12 mapped Hsf genes were found to possess conserved microsynteny among the species investigated, and Hsf orthologs were found in nine groups located in the single syntenic region compared with other legume species, but 10 other Hsfs in this species could not be localized on the genome, and their regions were not used for comparison. Therefore, the number of duplicated Hsfs that remain in *C. cajan* is uncertain.

### Strong purifying selection for Hsf genes in *G. max*

Almost the entire Hsf family of *G. max* has been expanded by two genome duplications. To better understand the evolutionary constraints acting on this gene family, we measured the Ka/Ks ratios for 35 unambiguous pairs of Hsf paralogs (not including paralogs from the flexible set) in the network of duplicated regions of *G. max*. The resulting pairwise comparison data showed that all the paralog pairs have Ka/Ks ratios <1 (Figure 3B), suggesting that the Hsf family has mainly undergone strong purifying selection, and the Hsf genes are slowly evolving at the protein level. Given the important role of the two rounds of whole-genome duplication in the evolution of the *G. max* Hsf family, the significance of changes in the strength of selection over evolutionary time was also stressed, and the Ka/Ks ratios were sorted into two sets on the basis of the Hsf paralogs that arose from either the recent or earlier whole-genome duplication. The average Ka/Ks ratio for the recent -duplicated Hsfs (0.30) was higher than that of the early-duplicated Hsfs (0.25), but there was no significant difference between these ratios (t-test, P>0.05). Moreover, the variance of the Ka/Ks ratios for the recent-duplicated Hsfs (0.013) was not significantly different from that of the early-duplicated Hsfs (0.006; F-test, P>0.05). This indicates that the younger and older proteins in the Hsf family are under similarly stable evolutionary constraints, which supports the notion that this family is essential for the regulation of cellular processes in *G. max*.

To assess the potential for selection on the regions surrounding Hsfs, pairwise Ka/Ks ratios were also calculated for the duplicated non-Hsf genes (flanking genes) between the duplicated regions containing Hsfs in *G. max*. Interestingly, all Ka/Ks values for 322 pairs of duplicated non-Hsf genes were lower than 1 (Figure 3C), clearly indicating that these genes are evolving under purifying selection. There was no significant difference in average Ka/Ks ratio between the recent-duplicated non-Hsf genes (0.29) and the early-duplicated non-Hsf genes (0.27; t-test, P>0.05). However, the variance of these ratios for the recent-duplicated non-Hsf genes (0.032) was significant greater than that for the early-duplicated ones (0.013; F-test, P<0.01). Duplicated non-Hsf genes have likely evolved in a more “dynamic” regime than that of the Hsf genes.

**Figure 6:**
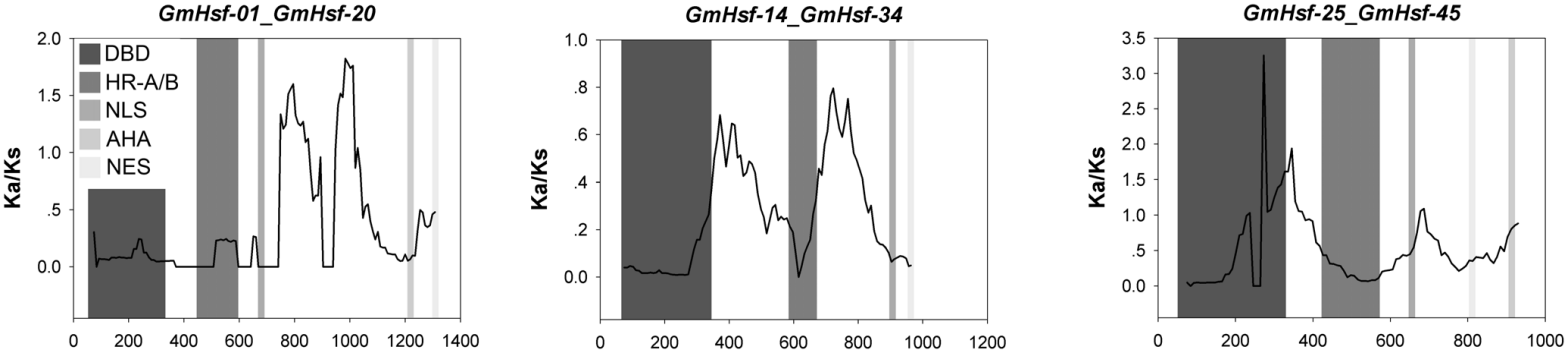
Sliding window plots of representative duplicated Hsf genes in *G. max*. As shown in the key, the gray blocks, from dark to light, indicate the positions of the DBD domain, HR-A/B region, NLS, NES and AHA motifs, respectively. The window size is 150 bp, and the step size is 9 bp. The data for all pairs of duplicated Hsf genes of soybean are shown in Figure S6.

Since positive selection at a few individual codon sites can be masked by overall strong purifying selection, we performed a sliding-window analysis of Ka/Ks between each pair of Hsf paralogs, which were derived from gene duplication events in *G. max*. As expected from the basic Ka/Ks analysis, sliding window analysis clearly showed that numerous sites/regions are under moderate to strong negative selection (Figure S6). As shown in Figure 6, the conserved domains of Hsfs, such as the DBD domains, HR-A/B regions and NLS motifs, are mainly subjected to strong purifying selection, with Ka/Ks ratios <<1. Moreover, the domains of Hsfs generally had lower Ka/Ka ratios (valleys) than the regions outside of them (peaks), which is consistent with functional constraint being dominant in these domains. There were a few exceptions to the generally low Ka/Ka ratios in domains. For instance, the comparison between *GmHsf-25* and *GmHsf-45* revealed sites with Ka/Ka ratios >>1 in the DBD domain, indicating positive selection in this region.

To further identify possible positive selection acting at specific sites, six site models that allow ω ratios to vary among sites were used based on the coding sequences of *G. max* Hsf family. To detect whether some sites along particular Hsf classes were under positive selection, the hypothesis testing on the class A and B *G. max* Hsfs was also performed by site models. In *G. max* Hsf family, the discrete model M3 fit better than the one-ratio model M0, suggesting that ω ratios vary among sites (Table S4; LRT, P<0.01). Both M1a–M2a and M7–M8 comparisons suggested that the most codon sites were under a strong constraint and no reliable positive selection sites were detected in *G. max* Hsf family (Table S4; LRT, P<0.01). In class A and B *G. max* Hsfs, M3 model also appears to be a better fit to the data than the M0 model, and the models M2a and M8 were not significantly better than the null hypothesis models M1a and M7 (Table S4; LRT, P<0.01). Only one positively selected site, listed in Table S4, was detected based on posterior probability in class A Hsfs of *G. max*. The results showed that *G. max* Hsf genes were highly conserved and the majority of sites were dominated by purifying selection.

### The expression patterns of Hsf genes in *L. japonicus*

**Figure 7:**
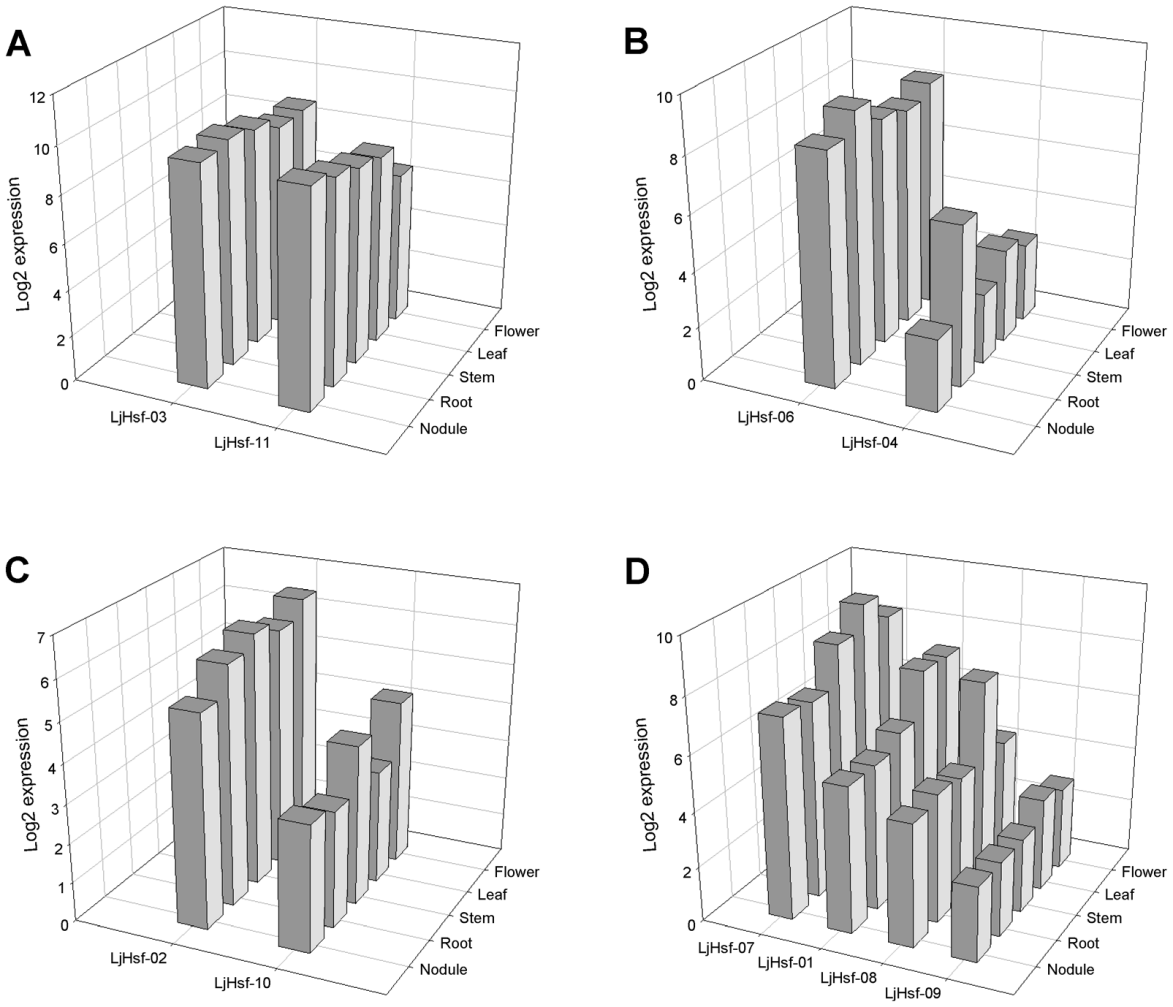
*L. japonicus* Hsf genes expression in various plant tissues. The type of tissue (nodule, root, stem, leaf and flower) and the gene name are shown on the y-axis and x-axis, respectively. Hierarchical clustering based on average log signal values in various tissues grouped 10 of the *L. japonicus* Hsf genes into four types (A–D).

In order to gain insight into the possible functions of Hsf genes, we comprehensively examined information about the expression of all *L. japonicus* Hsf genes using microarray data and quantitative real-time PCR analysis. We first analyzed the expression of *L. japonicus* Hsf genes in nodule, root, stem, leaf and flower from the microarray data (Figure 7). Out of 11 of these genes, the expression data for *LjHsf-05* were not included in the database. The ten remaining genes were expressed in all the tissues investigated, but they exhibited differential patterns in terms of both specificity and expression level. According to their expression profiles, *L. japonicus* Hsf genes can be classified into four types. The transcripts of the first type (*LjHsf-03* and *LjHsf-11*) were highly accumulated in both underground (nodule and root) and aerial (stem, leaf and flower) parts, but the expression level was higher in the underground parts (Figure 7A). In the second type, *LjHsf-04* showed maximum expression in the root and *LjHsf-06* had a similar pattern. However, *LjHsf-06* was much more highly expressed than *LjHsf-04* (Figure 7B). The genes of the third type (*LjHsf-02* and *LjHsf-10*) were expressed preferentially in stem and flower, and *LjHsf-02* showed higher expression than *LjHsf-10* (Figure 7C). The fourth type has four members (*LjHsf-01*, *LjHsf-07*, *LjHsf-08* and *LjHsf-09*), the genes predominantly expressed in leaves (Figure 7D). *LjHsf-01* and *LjHsf-09* were also expressed at higher levels in flowers than nodule, root and stem. Although the Hsf genes of the fourth type had similar expression pattern across a range of tissues, their transcript levels were quite diverse. *LjHsf-07* was the most highly expressed gene and *LjHsf-09* the lowest.

**Figure 8:**
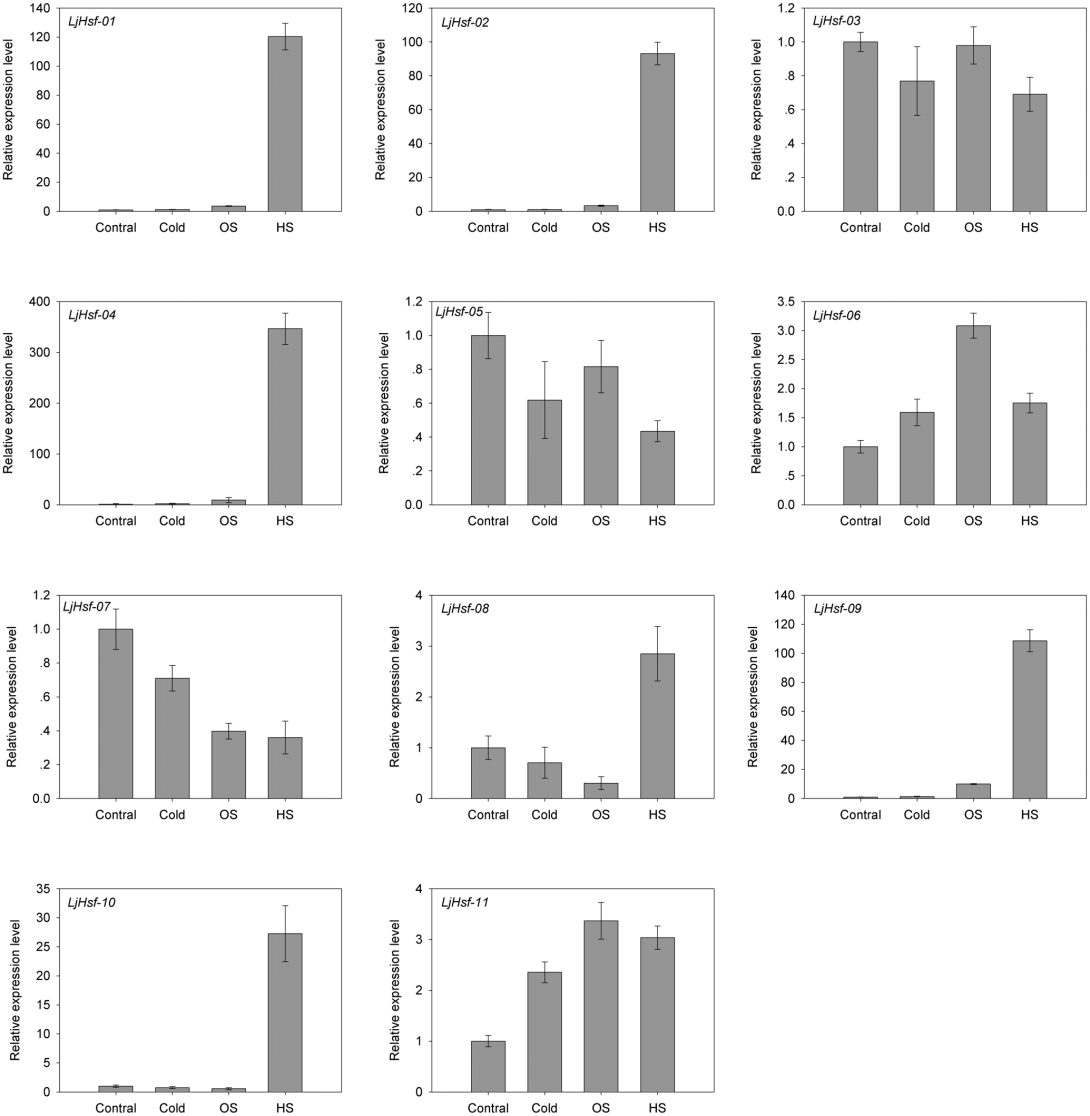
Expression of *L. japonicus* Hsf genes in response to abiotic stress measured by quantitative real-time PCR. The mRNA level of each gene in *L. japonicus* seedlings given heat (HS: 42°C), cold (4°C) and oxidative (OS: 10 mM H_2_O_2_) stress for 1 h was plotted relative to the value obtained for the unstressed contral. Error bars represent standard errors.

Quantitative real-time PCR analysis was then performed to evaluate the response of the *L. japonicus* Hsf gene family to abiotic stress. RNA was isolated from the leaves of 4-week-old *L. japonicus* seedlings subjected to heat, cold and H_2_O_2_ stress treatment and was used for the experiments. The results showed that a total of ten genes were significantly up- or down-regulated compared to controls (>2 or <0.5) in at least one of the stress conditions examined (Figure 8). Among these genes, most were responsive to more than one stress treatment. Two genes (*LjHsf-04* and *LjHsf-11*) were significantly up-regulated by all three stresses. Three (*LjHsf-01*, *LjHsf-02* and *LjHsf-09*) were expressed at remarkably high levels in response to both heat and H_2_O_2_ stress. *LjHsf-08* was induced by heat stress but was suppressed by H_2_O_2_ stress. *LjHsf-07* was significantly down-regulated upon exposure to heat and H_2_O_2_ stresses. A few genes were primarily responsive to one stress treatment. *LjHsf-05* and *LjHsf-10* responded specifically to heat stress, while *LjHsf-06* was distinctively up-regulated under H_2_O_2_ stress. In contrast, *LjHsf-03* showed minor fluctuations during all three stresses. It is worth noting that five genes (*LjHsf-01*, *LjHsf-02*, *LjHsf-04*, *LjHsf-09* and *LjHsf-10*) were strongly heat-inducible in our experiments, suggesting that they could have important roles in the heat shock regulatory network.

## Discussion

In this study, we identified 11, 19 and 13 Hsfs in the cool season legumes *L*. *japonicus*, *M*. *truncatula* and *C. arietinum*, respectively, and 46, 22 and 29 Hsfs in the tropical season legumes *G. max*, *C. cajan* and *P. vulgaris*, respectively. Before reconstructing the gene gain/loss history of legume Hsf families, it is necessary to trace the Hsf genes in different legume genomes back to a common ancestor. Phylogenetic trees are quite informative for inferring the number of Hsfs in the most recent common ancestor of the six legume species analyzed in this study [60]. There are 18 well-supported clades representing legume Hsfs (Figure 1), although the representatives of one or two species are missing from some clades. These clades are perceived as shared, and the genes in a shared clade are assumed to be descendants of an ancestral Hsf gene. Therefore, there are at least 18 Hsf genes in the most recent common ancestor of legumes. A large fraction of variability in the clades suggests lineage-specific gene gain and loss. For instance, in almost every clade, Hsfs from *G. max* are present in at least an extra copy compared with the other species, and the extra copy is very close to its potential paralog. These results are consistent with the well-documented fact that *G. max* has undergone an additional WGD not shared with by the other three species [8]. However, there are not simply twice as many Hsf genes in *G. max* vs. the other legumes, which indicates that differential gene loss events may have occurred in different species. Furthermore, all of the clades were confirmed by the following interspecies microsynteny analysis (except for the shared clade representing class C Hsfs), indicating most duplicates of class C Hsfs have been lost in legumes. When we compared the number of ancestral genes with those in the extant legumes species, it appeared as though the Hsf family had contracted in most of the cool season legumes and expanded in the tropical season legumes. For example, compared with the number of ancestral genes, the number of Hsf genes was nearly halved in *L*. *japonicus* but increased approximately 2.5-fold in *G. max*. These results most likely reflect the complex evolutionary history of the Hsf family in legume species.

In *G. max*, most Hsf genes were assigned to the duplicated segments, and these segments could be divided into two groups based on Ks values. The members of one group were supported with an average Ks value of 0.701, which corresponds to the second peak in Figure 3A. In *L*. *japonicus*, *M*. *truncatula* and *C. cajan*, a few Hsf-containing segments were matched in pairs. The Ks values for these pairs were included in Table 2, such as *MtHsf-03*/*MtHsf-16* (0.80) and *MtHsf-11*/*MtHsf-19* (0.77). All of these Ks values from *G. max* and other legumes probably represent the same polyploidy that occurred in all members of Papilionoideae subfamily approximately 59 Mya. Thus, the rate of synonymous substitution in *M. truncatula* appears to be greater than that in *G. max*. In a previous study of *Rxp* regions, higher rates of synonymous substitution were also detected in *M. truncatula* compared with *G. max* [61].

After the shared polyploidy event and following divergence from the other legumes, the lineage leading to present-day *G. max* is known to have undergone a second whole-genome duplication approximately 13 Mya; this duplication did not occur in *L*. *japonicus*, *M*. *truncatula* or other legume lineages. Two whole-genome duplications in the ancestor of *G. max* lead to the expectation of a maximum of four homoeologs in *G. max* genome [62], [63]. As illustrated in Figure 2, Hsf genes doubled twice in *G. max* and formed two pairs of paralogs, accordingly. In some cases, these four homoeologous Hsf regions were retained. For instance, *GmHsf-12*, *GmHsf-17*, *GmHsf-32* and *GmHsf-36* are located in four homoeologous regions, respectively. More conserved flanking protein-coding genes were identified between the two paralogous Hsf-containing segments derived from the recent duplication event than those from the ancient duplication (Table 2). This suggests that high levels of sequence conservation were maintained between recently duplicated regions in *G. max*, which is similar to previous observations [62], [64]. Furthermore, 17 pairs of Hsf genes of *G. max* generated by recent genome duplication were retained, while in eight pairs, one copy was lost (Figure 2). A higher, sharp peak was produced (the first peak in Figure 3A) when the number of gene pairs within these 17 pairs of recent duplicated regions was plotted against Ks values. These results indicate that many younger Hsf genes from recent genome duplications tended to be retained in *G. max* genome. By contrast, it has been demonstrated that massive gene losses occurred after tetraploidization in the maize ancestral lineage, and approximately 50% of the duplicated copies of genes have been removed or severely damaged over the past 12 million years [65], [66]. In *Glycine*, the large number of duplicate genes in homeologous regions indicates that the process of diploidization is a slow and ongoing process [67].

In light of the two above-mentioned rounds of genome duplication and the fact that genome duplication should double the number of genes, the *G. max* and other papilionoid legume genomes should show a 2∶1 relationship regarding the Hsf-containing regions. Each pair of *G. max* regions should have a corresponding orthologous region in *L. japonicas*, *M. truncatula* and *C. cajan*. In this study, by aligning Hsf-containing regions into paralogous pairs produced by ancient polyploidy, only one pair was detected in *L*. *japonicas* and *C. cajan*, with two pairs in *M*. *truncatula*. However, more than 90% of the chromosome regions hosting Hsf genes in *G. max* fell into pairs, triples or quadruples. Further analyses including estimates of the dates of duplications indicated that these duplicated regions arose from two polyploidy events in the *Glycine* lineage. A high degree of microsynteny between the genomes of the two model legumes has previously been found in a comparison of the genomic regions around the apyrase genes [68]. The analysis of microsynteny can help unravel the actual evolutionary relationships between Hsf regions among the legume species by taking advantage of the surrounding genomic sequences. When the Hsf-containing regions in *L. japonicas*, *M. truncatula* and *C. cajan* were compared to those in *G. max*, we found that significant synteny was maintained, although small insertions/deletions and inversions were observed between regions (Figure 4 and Figure S5). Almost all Hsf-containing regions in *L. japonicas*, *M. truncatula* and *C. cajan* showed close relationships with the orthologous duplicated regions in *G. max*. Syntenic regions are thought to share a common origin derived from ancient legume duplication. In most cases, a single region of *L*. *japonicus*, *M*. *truncatula* and *C. cajan* was syntenic to two or three duplicated regions in *G. max*, and in many cases, two paralogons appeared to be missing in *L*. *japonicus* or *M*. *truncatula*. This indicates that the duplicated copies of Hsf genes in these genomes have been removed or severely damaged after the genome duplications occurred. Approximately half of the Hsf genes of *C. cajan* were not included in the map-based analyses because these genes could not be localized to the genome owing to their unknown chromosome positions. These results may therefore not hold true for *C. cajan*.

One possible mechanism behind this phenomenon, that many Hsf genes produced by the ancient genome duplication have been lost in *L*. *japonicus* and *M*. *truncatula*, is diploidization following the early legume genome duplication. Previous studies have demonstrated that there was substantially less conservation within internal duplications in either *M*. *truncatula* or *L*. *japonicus* than in synteny blocks between the two genomes [5]. The fate of these duplicate genes is more likely to be under the control of natural selection (nonrandom loss) than for genes that are not dosage dependant [69].

Duplicated genes undergo a short period of shared “relaxed” selection during their early evolutionary lives, evolving in a neutral way. Following this selection, most paralogs are lost within a few million years, and only a few paralogs are preserved and undergo purifying selection [10], [53], [70]. Although many gene loss events were found to have occurred in legume Hsf families, two Hsf regions of *L*. *japonicus* (*LjHsf-06*/*LjHsf-11*) and the two Hsf regions of *M*. *truncatula* (*MtHsf-03*/*MtHsf-16*) were found to have conserved microsynteny to the four Hsf regions of *G. max* (*GmHsf-12*/*GmHsf-17*/*GmHsf-32*/*GmHsf-36*; Figure 5B). This suggests that these sets of Hsf genes may perform a basic, important role in legumes and have remained intact after genome duplications.

The observation that multiple copies of Hsf were retained in *G. max* is reasonable from an evolutionary perspective because Hsfs confer various abiotic and biotic resistance traits to plants [71]–[77]. The dosage of protein may have increased due to the presence of numerous Hsf genes, thereby leading to a corresponding increase in resistance. Previous studies have demonstrated the advantages of increased dosage of genes involved in plant resistance, for instance, resistance to glyphosate in plants [78]. Furthermore, strong purifying selection was detected between the paralogs produced from the recent genome duplication and the ancient duplication in *G. max*. Purifying selection probably plays a key role in maintaining the long-term stability of biological structures of *G. max* Hsfs by removing deleterious mutations, thus ensuring that gene functions are maintained as long as they are needed.

Moreover, *G. max* is a member of tropical season legumes; natural selection may have played a role in determining the number of duplicates within the Hsf gene family in the tropical season legumes, which are better adapted to more tropical climates. On the contrary, *L. japonicus* and *M. truncatula* are cool season legumes, and they may therefore require fewer Hsf genes. Thus, many copies of these genes may have been lost during the long-term evolutionary process.

Although the legume species differ in genome size, basic chromosome number and ploidy level, comparative genomics can be used for a bridging model with other legume species in view of their close phylogenetic relationships and extensive genome conversation [79]. *L. japonicus* was selected as a model system for gene function characterization because of its small genome size and improving knockout and over-expression techniques, and efforts were made to translate information gained from it into exploration to other legume species. *L. japonicus* maintained only 11 Hsf genes and this number is probably the lowest among the reported Hsf gene families of higher plants. In our study, the orthologs of the 11 Hsf genes were unambiguously defined in the other legumes through microsyntenic analysis. These directed our interest towards further understanding of the expression characteristics of these Hsf genes in different tissues and their responses to abiotic stresses. In several plants, Hsf gene expression has been found to be tissue- and stage-specific [80]–[82]. Our study revealed that none of the *L. japonicus* Hsf genes examined was expressed only in a particular tissue type, suggesting they play regulatory roles at multiple developmental stages; nevertheless, selected *L. japonicus* Hsf genes exhibited higher levels of expression in particular tissue types, indicating that members of this family might take part in different biological processes in this species. In particular, the member of class B and the member of class A showed similar expression patterns in tissues, e.g. *LjHsf-02* and *LjHsf-10* were expressed at a higher level in stem and flower than other tissues, supporting the assumption that they could co-operate with each other. In *Arabidopsis* and rice, the expression of Hsf genes was strongly induced by heat, cold, salt and osmotic stress [81], [83]. In our study, we also found that the Hsf genes of *L. japonicus* were responsive to diverse abiotic stresses, and heat induced their expression more strongly than oxidation and cold. *LjHsf-01* of subclass A2, *LjHsf-02* of subclass B2 and *LjHsf-04* of subclass B1 were strongly and transiently up-regulated by heat shock. Among all Hsf genes in *Arabidopsis*, *HsfA2* was most highly expressed under high temperature conditions and was identified as a key positive regulator of the heat stress response [84], [85]. *Arabidopsis HsfB1* and *HsfB2b* repressed the general heat shock response in the absence of excessive heat but were necessary for the development of acquired thermotolerance under heat stress conditions [86]. It is noteworthy that many Hsf genes of *L. japonicus* induced by heat stress were also induced by oxidative stress, and *LjHsf-04* and *LjHsf-11* were induced by all three stresses tested. These findings could support the notion that Hsfs serve as important sensors for H_2_O_2_ in plants and could be pivotal in linking the heat shock response with other stress-responsive signaling networks [87]. The expression pattern described can provide a basis for identifying the roles of the retained members of the *L. japonicus* Hsf family, and would give clues for studying their syntenic counterparts in other legume species.

## Supporting Information

Figure S1 **DBD domain alignment of Hsf proteins from** ***L. japonicus*** **,** ***M. truncatula*** **,** ***C. arietinum*** **,** ***G. max*** **,** ***C. cajan*** **,** ***P. vulgaris*** **and three lower plants.**
(PDF)

Figure S2 **HR-A/B region alignment of Hsf proteins from** ***L. japonicus*** **,** ***M. truncatula*** **,** ***C. arietinum*** **,** ***G. max*** **,** ***C. cajan*** **,** ***P. vulgaris*** **and three lower plants.**
(PDF)

Figure S3 **NJ phylogeny of** ***A. thaliana*** **,** ***O. sativa*** **,** ***Z. mays*** **,** ***L. japonicus*** **,** ***M. truncatula*** **,** ***C. arietinum*** **,** ***G. max*** **,** ***C. cajan*** **and** ***P. vulgaris*** **Hsf proteins.**
(PDF)

Figure S4 **ML phylogeny of** ***L. japonicus*** **,** ***M. truncatula*** **,** ***C. arietinum*** **,** ***G. max*** **,** ***C. cajan*** **and** ***P. vulgaris*** **Hsf proteins.**
(PDF)

Figure S5 **The full syntenic maps of chromosome regions containing Hsf genes across** ***L. japonicus*** **,** ***M. truncatula*** **,** ***G. max*** **and** ***C. cajan*** **genes.**
(PDF)

Figure S6 **Sliding window analysis employed to estimate selective pressures on 35 pairs of duplicated Hsf genes in** ***G. max*** **.**
(PDF)

Table S1 **Primers used in quantitative real-time PCR.**
(PDF)

Table S2 **Information about Hsfs in** ***L. japonicus*** **,** ***M. truncatula*** **,** ***C. arietinum*** **,** ***G. max*** **,** ***C. cajan*** **,** ***P. vulgaris*** **,** ***S. moellendorffii*** **,** ***P. patens*** **and** ***C. reinhardtii*** **.**
(PDF)

Table S3 **Domain and motif survey of legume and three lower plant Hsfs.**
(PDF)

Table S4 **Likelihood ratio tests and parameter estimations for the six site models based on the coding sequences of** ***G. max*** **Hsf genes.**
(PDF)

## References

[1] Cui L, Wall PK, Leebens-Mack JH, Lindsay BG, Soltis DE, et al (2006) Widespread genome duplications throughout the history of flowering plants. Genome Res 16: 738–749. 16702410 10.1101/gr.4825606 PMC1479859

[2] Jaillon O, Aury JM, Noel B, Policriti A, Clepet C, et al (2007) The grapevine genome sequence suggests ancestral hexaploidization in major angiosperm phyla. Nature 449: 463–467. 17721507 10.1038/nature06148

[3] Blanc G, Wolfe KH (2004) Widespread paleopolyploidy in model plant species inferred from age distributions of duplicate genes. Plant Cell 16: 1667–1678. 15208399 10.1105/tpc.021345 PMC514152

[4] Adams KL, Wendel JF (2005) Polyploidy and genome evolution in plants. Curr Opin Plant Biol 8: 135–141. 15752992 10.1016/j.pbi.2005.01.001

[5] Cannon SB, Sterck L, Rombauts S, Sato S, Cheung F, et al (2006) Legume genome evolution viewed through the Medicago truncatula and Lotus japonicus genomes. Proceedings of the National Academy of Sciences 103: 14959–14964. 10.1073/pnas.0603228103 PMC1578499 17003129

[6] Fawcett JA, Maere S, Van de Peer Y (2009) Plants with double genomes might have had a better chance to survive the Cretaceous-Tertiary extinction event. Proc Natl Acad Sci U S A 106: 5737–5742. 19325131 10.1073/pnas.0900906106 PMC2667025

[7] Cannon SB, Ilut D, Farmer AD, Maki SL, May GD, et al (2010) Polyploidy did not predate the evolution of nodulation in all legumes. PLoS One 5: e11630. 20661290 10.1371/journal.pone.0011630 PMC2905438

[8] Schmutz J, Cannon SB, Schlueter J, Ma J, Mitros T, et al (2010) Genome sequence of the palaeopolyploid soybean. NATURE 463: 178–183. 20075913 10.1038/nature08670

[9] Young ND, Bharti AK (2012) Genome-enabled insights into legume biology. Annual review of plant biology 63: 283–305. 10.1146/annurev-arplant-042110-103754 22404476

[10] De Grassi A, Lanave C, Saccone C (2008) Genome duplication and gene-family evolution: the case of three OXPHOS gene families. Gene 421: 1–6. 18573316 10.1016/j.gene.2008.05.011

[11] Li J, Ding J, Zhang W, Zhang Y, Tang P, et al (2010) Unique evolutionary pattern of numbers of gramineous NBS–LRR genes. Molecular Genetics and Genomics 283: 427–438. 20217430 10.1007/s00438-010-0527-6

[12] Cheng Y, Li X, Jiang H, Ma W, Miao W, et al (2012) Systematic analysis and comparison of nucleotide-binding site disease resistance genes in maize. FEBS J 279: 2431–2443. 22564701 10.1111/j.1742-4658.2012.08621.x

[13] Cannon SB, Mitra A, Baumgarten A, Young ND, May G (2004) The roles of segmental and tandem gene duplication in the evolution of large gene families in Arabidopsis thaliana. BMC Plant Biol 4: 10. 15171794 10.1186/1471-2229-4-10 PMC446195

[14] Maere S, De Bodt S, Raes J, Casneuf T, Van Montagu M, et al (2005) Modeling gene and genome duplications in eukaryotes. Proc Natl Acad Sci U S A 102: 5454–5459. 15800040 10.1073/pnas.0501102102 PMC556253

[15] Chapman BA, Bowers JE, Feltus FA, Paterson AH (2006) Buffering of crucial functions by paleologous duplicated genes may contribute cyclicality to angiosperm genome duplication. Proc Natl Acad Sci U S A 103: 2730–2735. 16467140 10.1073/pnas.0507782103 PMC1413778

[16] Baniwal SK, Bharti K, Chan KY, Fauth M, Ganguli A, et al (2004) Heat stress response in plants: a complex game with chaperones and more than twenty heat stress transcription factors. J Biosci 29: 471–487. 15625403 10.1007/BF02712120

[17] Kotak S, Larkindale J, Lee U, von Koskull-Doring P, Vierling E, et al (2007) Complexity of the heat stress response in plants. Curr Opin Plant Biol 10: 310–316. 17482504 10.1016/j.pbi.2007.04.011

[18] Morimoto RI (1998) Regulation of the heat shock transcriptional response: cross talk between a family of heat shock factors, molecular chaperones, and negative regulators. Genes Dev 12: 3788–3796. 9869631 10.1101/gad.12.24.3788

[19] Nover L, Scharf KD (1997) Heat stress proteins and transcription factors. Cell Mol Life Sci 53: 80–103. 9118000 10.1007/PL00000583 PMC11488844

[20] Schoffl F, Prandl R, Reindl A (1998) Regulation of the heat-shock response. Plant Physiol 117: 1135–1141. 9701569 10.1104/pp.117.4.1135 PMC1539185

[21] Wu C (1995) Heat shock transcription factors: structure and regulation. Annu Rev Cell Dev Biol 11: 441–469. 8689565 10.1146/annurev.cb.11.110195.002301

[22] Scharf KD, Berberich T, Ebersberger I, Nover L (2012) The plant heat stress transcription factor (Hsf) family: structure, function and evolution. Biochim Biophys Acta 1819: 104–119. 22033015 10.1016/j.bbagrm.2011.10.002

[23] Lutz N (1987) Expression of heat shock genes in homologous and heterologous systems. Enzyme and Microbial Technology 9: 130–144.

[24] Peteranderl R, Rabenstein M, Shin YK, Liu CW, Wemmer DE, et al (1999) Biochemical and biophysical characterization of the trimerization domain from the heat shock transcription factor. Biochemistry 38: 3559–3569. 10090742 10.1021/bi981774j

[25] Doring P, Treuter E, Kistner C, Lyck R, Chen A, et al (2000) The role of AHA motifs in the activator function of tomato heat stress transcription factors HsfA1 and HsfA2. Plant Cell 12: 265–278. 10662862 PMC139763

[26] Kotak S, Port M, Ganguli A, Bicker F, von Koskull-Doring P (2004) Characterization of C-terminal domains of Arabidopsis heat stress transcription factors (Hsfs) and identification of a new signature combination of plant class A Hsfs with AHA and NES motifs essential for activator function and intracellular localization. Plant J 39: 98–112. 15200645 10.1111/j.1365-313X.2004.02111.x

[27] Clos J, Westwood JT, Becker PB, Wilson S, Lambert K, et al (1990) Molecular cloning and expression of a hexameric Drosophila heat shock factor subject to negative regulation. Cell 63: 1085–1097. 2257625 10.1016/0092-8674(90)90511-c

[28] Hsu AL, Murphy CT, Kenyon C (2003) Regulation of aging and age-related disease by DAF-16 and heat-shock factor. Science 300: 1142–1145. 12750521 10.1126/science.1083701

[29] Sorger PK, Pelham HR (1988) Yeast heat shock factor is an essential DNA-binding protein that exhibits temperature-dependent phosphorylation. Cell 54: 855–864. 3044613 10.1016/s0092-8674(88)91219-6

[30] Wiederrecht G, Seto D, Parker CS (1988) Isolation of the gene encoding the S. cerevisiae heat shock transcription factor. Cell 54: 841–853. 3044612 10.1016/s0092-8674(88)91197-x

[31] Xiao X, Zuo X, Davis AA, McMillan DR, Curry BB, et al (1999) HSF1 is required for extra-embryonic development, postnatal growth and protection during inflammatory responses in mice. EMBO J 18: 5943–5952. 10545106 10.1093/emboj/18.21.5943 PMC1171660

[32] Xing H, Wilkerson DC, Mayhew CN, Lubert EJ, Skaggs HS, et al (2005) Mechanism of hsp70i gene bookmarking. Science 307: 421–423. 15662014 10.1126/science.1106478

[33] Fujimoto M, Izu H, Seki K, Fukuda K, Nishida T, et al (2004) HSF4 is required for normal cell growth and differentiation during mouse lens development. EMBO J 23: 4297–4306. 15483628 10.1038/sj.emboj.7600435 PMC524399

[34] Nover L, Bharti K, Doring P, Mishra SK, Ganguli A, et al (2001) Arabidopsis and the heat stress transcription factor world: how many heat stress transcription factors do we need? Cell Stress Chaperones 6: 177–189. 11599559 10.1379/1466-1268(2001)006<0177:aathst>2.0.co;2 PMC434399

[35] Guo J, Wu J, Ji Q, Wang C, Luo L, et al (2008) Genome-wide analysis of heat shock transcription factor families in rice and Arabidopsis. J Genet Genomics 35: 105–118. 18407058 10.1016/S1673-8527(08)60016-8

[36] Xiong Y, Liu T, Tian C, Sun S, Li J, et al (2005) Transcription factors in rice: a genome-wide comparative analysis between monocots and eudicots. Plant Mol Biol 59: 191–203. 16217612 10.1007/s11103-005-6503-6

[37] Lin YX, Jiang HY, Chu ZX, Tang XL, Zhu SW, et al (2011) Genome-wide identification, classification and analysis of heat shock transcription factor family in maize. BMC Genomics 12: 76. 21272351 10.1186/1471-2164-12-76 PMC3039612

[38] Garg R, Patel RK, Jhanwar S, Priya P, Bhattacharjee A, et al (2011) Gene discovery and tissue-specific transcriptome analysis in chickpea with massively parallel pyrosequencing and web resource development. Plant Physiol 156: 1661–1678. 21653784 10.1104/pp.111.178616 PMC3149962

[39] Czarnecka-Verner E, Yuan CX, Fox PC, Gurley WB (1995) Isolation and characterization of six heat shock transcription factor cDNA clones from soybean. Plant Mol Biol 29: 37–51. 7579166 10.1007/BF00019117

[40] Punta M, Coggill PC, Eberhardt RY, Mistry J, Tate J, et al (2012) The Pfam protein families database. Nucleic Acids Res 40: D290–301. 22127870 10.1093/nar/gkr1065 PMC3245129

[41] Letunic I, Doerks T, Bork P (2012) SMART 7: recent updates to the protein domain annotation resource. Nucleic Acids Res 40: D302–305. 22053084 10.1093/nar/gkr931 PMC3245027

[42] Delorenzi M, Speed T (2002) An HMM model for coiled-coil domains and a comparison with PSSM-based predictions. Bioinformatics 18: 617–625. 12016059 10.1093/bioinformatics/18.4.617

[43] Cokol M, Nair R, Rost B (2000) Finding nuclear localization signals. EMBO Rep 1: 411–415. 11258480 10.1093/embo-reports/kvd092 PMC1083765

[44] la Cour T, Kiemer L, Molgaard A, Gupta R, Skriver K, et al (2004) Analysis and prediction of leucine-rich nuclear export signals. Protein Eng Des Sel 17: 527–536. 15314210 10.1093/protein/gzh062

[45] Thompson JD, Gibson TJ, Plewniak F, Jeanmougin F, Higgins DG (1997) The CLUSTAL_X windows interface: flexible strategies for multiple sequence alignment aided by quality analysis tools. Nucleic acids research 25: 4876–4882. 9396791 10.1093/nar/25.24.4876 PMC147148

[46] Tamura K, Dudley J, Nei M, Kumar S (2007) MEGA4: Molecular Evolutionary Genetics Analysis (MEGA) software version 4.0. Mol Biol Evol 24: 1596–1599. 17488738 10.1093/molbev/msm092

[47] Hanada K, Zou C, Lehti-Shiu MD, Shinozaki K, Shiu SH (2008) Importance of lineage-specific expansion of plant tandem duplicates in the adaptive response to environmental stimuli. Plant Physiol 148: 993–1003. 18715958 10.1104/pp.108.122457 PMC2556807

[48] Maher C, Stein L, Ware D (2006) Evolution of Arabidopsis microRNA families through duplication events. Genome research 16: 510–519. 16520461 10.1101/gr.4680506 PMC1457037

[49] Zhang X, Feng Y, Cheng H, Tian D, Yang S, et al (2011) Relative evolutionary rates of NBS-encoding genes revealed by soybean segmental duplication. Mol Genet Genomics 285: 79–90. 21080199 10.1007/s00438-010-0587-7

[50] Sato S, Nakamura Y, Kaneko T, Asamizu E, Kato T, et al (2008) Genome structure of the legume, Lotus japonicus. DNA Res 15: 227–239. 18511435 10.1093/dnares/dsn008 PMC2575887

[51] Edgar RC (2004) MUSCLE: multiple sequence alignment with high accuracy and high throughput. Nucleic Acids Res 32: 1792–1797. 15034147 10.1093/nar/gkh340 PMC390337

[52] Suyama M, Torrents D, Bork P (2006) PAL2NAL: robust conversion of protein sequence alignments into the corresponding codon alignments. Nucleic acids research 34: W609–W612. 16845082 10.1093/nar/gkl315 PMC1538804

[53] Lynch M, Conery JS (2000) The evolutionary fate and consequences of duplicate genes. Science 290: 1151–1155. 11073452 10.1126/science.290.5494.1151

[54] Yang Z (2007) PAML 4: phylogenetic analysis by maximum likelihood. Mol Biol Evol 24: 1586–1591. 17483113 10.1093/molbev/msm088

[55] Yang Z, Nielsen R, Goldman N, Pedersen AM (2000) Codon-substitution models for heterogeneous selection pressure at amino acid sites. Genetics 155: 431–449. 10790415 10.1093/genetics/155.1.431 PMC1461088

[56] Yang Z, Wong WS, Nielsen R (2005) Bayes empirical bayes inference of amino acid sites under positive selection. Mol Biol Evol 22: 1107–1118. 15689528 10.1093/molbev/msi097

[57] Hogslund N, Radutoiu S, Krusell L, Voroshilova V, Hannah MA, et al (2009) Dissection of symbiosis and organ development by integrated transcriptome analysis of lotus japonicus mutant and wild-type plants. PLoS One 4: e6556. 19662091 10.1371/journal.pone.0006556 PMC2717213

[58] Eisen MB, Spellman PT, Brown PO, Botstein D (1998) Cluster analysis and display of genome-wide expression patterns. Proc Natl Acad Sci U S A 95: 14863–14868. 9843981 10.1073/pnas.95.25.14863 PMC24541

[59] Bertioli DJ, Moretzsohn MC, Madsen LH, Sandal N, Leal-Bertioli SC, et al (2009) An analysis of synteny of Arachis with Lotus and Medicago sheds new light on the structure, stability and evolution of legume genomes. BMC Genomics 10: 45. 19166586 10.1186/1471-2164-10-45 PMC2656529

[60] Nam J, Kim J, Lee S, An G, Ma H, et al (2004) Type I MADS-box genes have experienced faster birth-and-death evolution than type II MADS-box genes in angiosperms. Proceedings of the National Academy of Sciences of the United States of America 101: 1910. 14764899 10.1073/pnas.0308430100 PMC357026

[61] Kim KD, Shin JH, Van K, Kim DH, Lee SH (2009) Dynamic rearrangements determine genome organization and useful traits in soybean. Plant Physiol 151: 1066–1076. 19684227 10.1104/pp.109.141739 PMC2773080

[62] Innes RW, Ameline-Torregrosa C, Ashfield T, Cannon E, Cannon SB, et al (2008) Differential accumulation of retroelements and diversification of NB-LRR disease resistance genes in duplicated regions following polyploidy in the ancestor of soybean. Plant Physiol 148: 1740–1759. 18842825 10.1104/pp.108.127902 PMC2593655

[63] Shin JH, Van K, Kim DH, Kim KD, Jang YE, et al (2008) The lipoxygenase gene family: a genomic fossil of shared polyploidy between Glycine max and Medicago truncatula. BMC Plant Biol 8: 133. 19105811 10.1186/1471-2229-8-133 PMC2644698

[64] Van K, Kim DH, Cai CM, Kim MY, Shin JH, et al (2008) Sequence level analysis of recently duplicated regions in soybean [Glycine max (L.) Merr.] genome. DNA Res 15: 93–102. 18334514 10.1093/dnares/dsn001 PMC2650623

[65] Lai J, Ma J, Swigonova Z, Ramakrishna W, Linton E, et al (2004) Gene loss and movement in the maize genome. Genome Res 14: 1924–1931. 15466290 10.1101/gr.2701104 PMC524416

[66] Messing J, Bharti AK, Karlowski WM, Gundlach H, Kim HR, et al (2004) Sequence composition and genome organization of maize. Proc Natl Acad Sci U S A 101: 14349–14354. 15388850 10.1073/pnas.0406163101 PMC521949

[67] Schlueter JA, Scheffler BE, Schlueter SD, Shoemaker RC (2006) Sequence conservation of homeologous bacterial artificial chromosomes and transcription of homeologous genes in soybean (Glycine max L. Merr.). Genetics 174: 1017–1028. 16888343 10.1534/genetics.105.055020 PMC1602103

[68] Cannon SB, McCombie WR, Sato S, Tabata S, Denny R, et al (2003) Evolution and microsynteny of the apyrase gene family in three legume genomes. Mol Genet Genomics 270: 347–361. 14598165 10.1007/s00438-003-0928-x

[69] Blanc G, Wolfe KH (2004) Functional divergence of duplicated genes formed by polyploidy during Arabidopsis evolution. Plant Cell 16: 1679–1691. 15208398 10.1105/tpc.021410 PMC514153

[70] Lynch M, Conery JS (2003) The evolutionary demography of duplicate genes. J Struct Funct Genomics 3: 35–44. 12836683

[71] Lohmann C, Eggers-Schumacher G, Wunderlich M, Schoffl F (2004) Two different heat shock transcription factors regulate immediate early expression of stress genes in Arabidopsis. Mol Genet Genomics 271: 11–21. 14655047 10.1007/s00438-003-0954-8

[72] Charng Y, Liu H, Liu N, Chi W, Wang C, et al (2007) A heat-inducible transcription factor, HsfA2, is required for extension of acquired thermotolerance in Arabidopsis. Plant Physiology 143: 251. 17085506 10.1104/pp.106.091322 PMC1761974

[73] Davletova S, Rizhsky L, Liang H, Shengqiang Z, Oliver DJ, et al (2005) Cytosolic ascorbate peroxidase 1 is a central component of the reactive oxygen gene network of Arabidopsis. The Plant Cell Online 17: 268–281. 10.1105/tpc.104.026971 PMC544504 15608336

[74] Sakuma Y, Maruyama K, Qin F, Osakabe Y, Shinozaki K, et al (2006) Dual function of an Arabidopsis transcription factor DREB2A in water-stress-responsive and heat-stress-responsive gene expression. Proceedings of the National Academy of Sciences 103: 18822. 10.1073/pnas.0605639103 PMC1693746 17030801

[75] Ogawa D, Yamaguchi K, Nishiuchi T (2007) High-level overexpression of the Arabidopsis HsfA2 gene confers not only increased themotolerance but also salt/osmotic stress tolerance and enhanced callus growth. Journal of experimental botany 58: 3373. 17890230 10.1093/jxb/erm184

[76] Kumar M, Busch W, Birke H, Kemmerling B, Nurnberger T, et al (2009) Heat shock factors HsfB1 and HsfB2b are involved in the regulation of Pdf1.2 expression and pathogen resistance in Arabidopsis. Mol Plant 2: 152–165. 19529832 10.1093/mp/ssn095 PMC2639743

[77] Czarnecka-Verner E, Pan S, Salem T, Gurley WB (2004) Plant class B HSFs inhibit transcription and exhibit affinity for TFIIB and TBP. Plant Mol Biol 56: 57–75. 15604728 10.1007/s11103-004-2307-3

[78] Widholm JM, Chinnala AR, Ryu JH, Song HS, Eggett T, et al (2001) Glyphosate selection of gene amplification in suspension cultures of 3 plant species. Physiol Plant 112: 540–545. 11473714 10.1034/j.1399-3054.2001.1120411.x

[79] Zhu H, Choi HK, Cook DR, Shoemaker RC (2005) Bridging model and crop legumes through comparative genomics. Plant Physiol 137: 1189–1196. 15824281 10.1104/pp.104.058891 PMC1088312

[80] Kotak S, Vierling E, Baumlein H, von Koskull-Doring P (2007) A novel transcriptional cascade regulating expression of heat stress proteins during seed development of Arabidopsis. Plant Cell 19: 182–195. 17220197 10.1105/tpc.106.048165 PMC1820961

[81] Swindell WR, Huebner M, Weber AP (2007) Transcriptional profiling of Arabidopsis heat shock proteins and transcription factors reveals extensive overlap between heat and non-heat stress response pathways. BMC Genomics 8: 125. 17519032 10.1186/1471-2164-8-125 PMC1887538

[82] Giorno F, Wolters-Arts M, Grillo S, Scharf KD, Vriezen WH, et al (2010) Developmental and heat stress-regulated expression of HsfA2 and small heat shock proteins in tomato anthers. J Exp Bot 61: 453–462. 19854799 10.1093/jxb/erp316 PMC2803211

[83] Mittal D, Chakrabarti S, Sarkar A, Singh A, Grover A (2009) Heat shock factor gene family in rice: genomic organization and transcript expression profiling in response to high temperature, low temperature and oxidative stresses. Plant Physiol Biochem 47: 785–795. 19539489 10.1016/j.plaphy.2009.05.003

[84] Nishizawa A, Yabuta Y, Yoshida E, Maruta T, Yoshimura K, et al (2006) Arabidopsis heat shock transcription factor A2 as a key regulator in response to several types of environmental stress. The Plant Journal 48: 535–547. 17059409 10.1111/j.1365-313X.2006.02889.x

[85] Schramm F, Ganguli A, Kiehlmann E, Englich G, Walch D, et al (2006) The heat stress transcription factor HsfA2 serves as a regulatory amplifier of a subset of genes in the heat stress response in Arabidopsis. Plant molecular biology 60: 759–772. 16649111 10.1007/s11103-005-5750-x

[86] Ikeda M, Mitsuda N, Ohme-Takagi M (2011) Arabidopsis HsfB1 and HsfB2b Act as Repressors of the Expression of Heat-Inducible Hsfs But Positively Regulate the Acquired Thermotolerance. Plant Physiology 157: 1243–1254. 21908690 10.1104/pp.111.179036 PMC3252156

[87] Miller G, Shulaev V, Mittler R (2008) Reactive oxygen signaling and abiotic stress. Physiol Plant 133: 481–489. 18346071 10.1111/j.1399-3054.2008.01090.x

