# Supplementary figures and images for: Genome Duplication and Gene Loss Affect the Evolution of Heat Shock Transcription Factor Genes in Legumes
Source: PLoS One. 2014 Jul 21;9(7):e102825. doi: 10.1371/journal.pone.0102825 (PMC4105503; doi:10.1371/journal.pone.0102825)

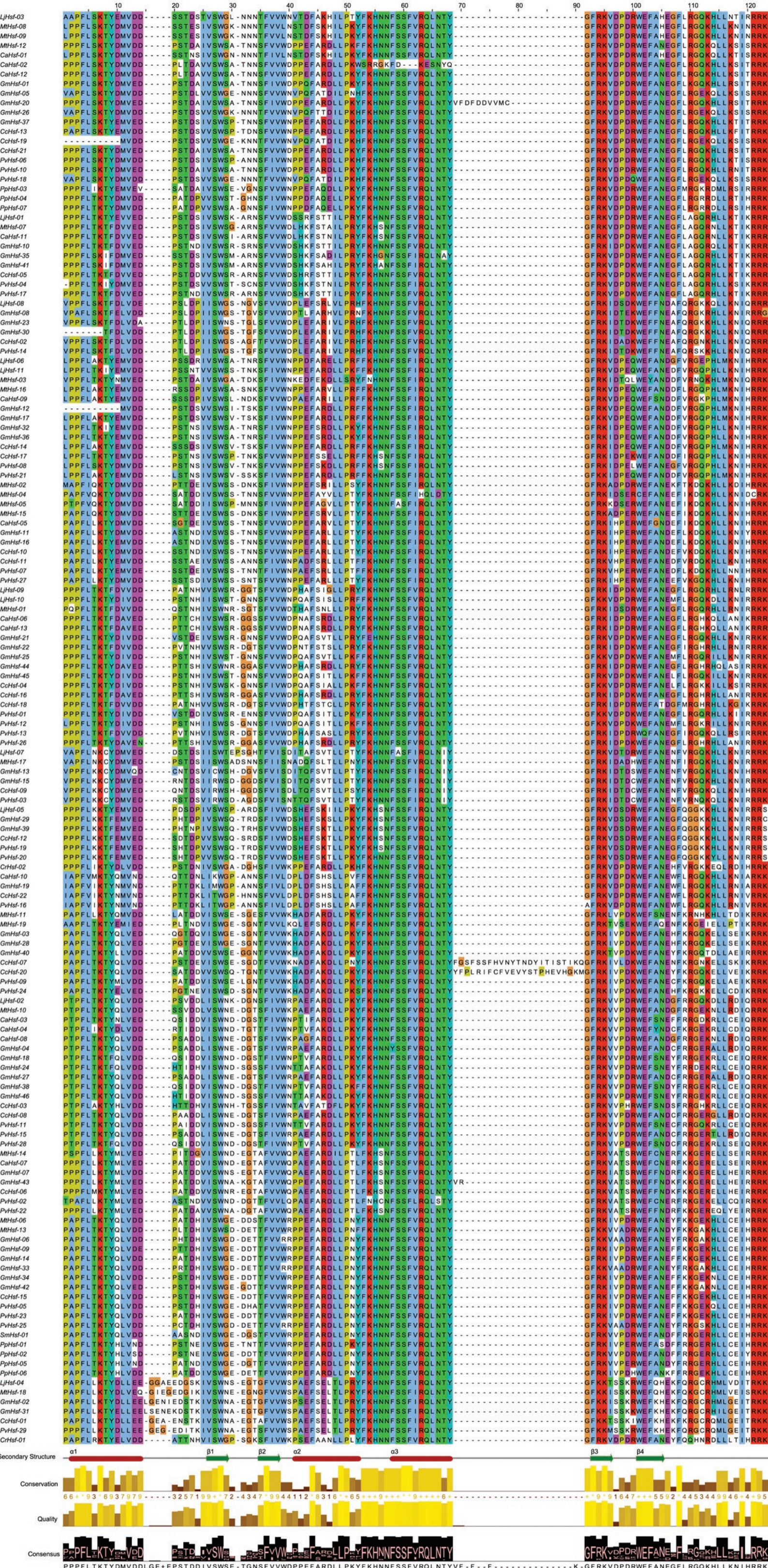

Supplement: Figure S1 — DBD domain alignment of Hsf proteins from L. japonicus , M. truncatula , C. arietinum , G. max , C. cajan , P. vulgaris and three lower plants. (PDF) [file pone.0102825.s001.pdf]

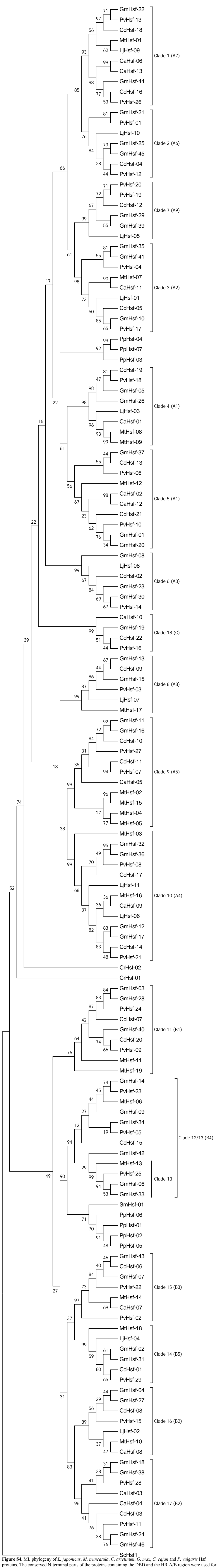

Supplement: Figure S4 — ML phylogeny of L. japonicus , M. truncatula , C. arietinum , G. max , C. cajan and P. vulgaris Hsf proteins. (PDF) [file pone.0102825.s004.pdf]
