# Supplementary material for: Genome Duplication and Gene Loss Affect the Evolution of Heat Shock Transcription Factor Genes in Legumes
Source: PLoS One. 2014 Jul 21;9(7):e102825. doi: 10.1371/journal.pone.0102825 (PMC4105503; doi:10.1371/journal.pone.0102825)
Supplement: Figure S2 — HR-A/B region alignment of Hsf proteins from L. japonicus , M. truncatula , C. arietinum , G. max , C. cajan , P. vulgaris and three lower plants. (PDF) [file pone.0102825.s002.pdf]

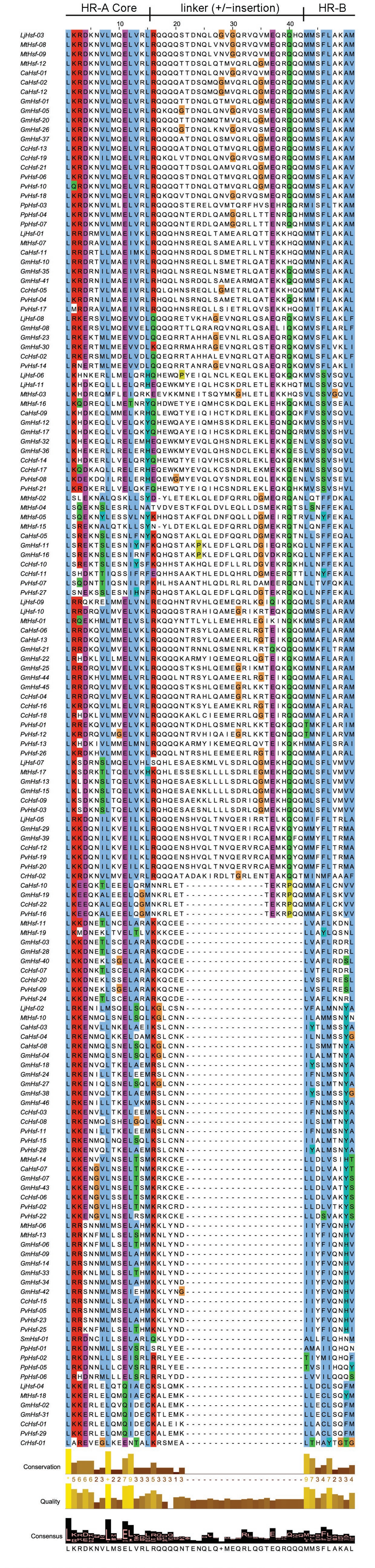


**Figure S2.** HR-A/B region alignment of Hsf proteins from *L. japonicus*, *M. truncatula*, *C. arietinum*, *G. max*, *C. cajan*, *P. vulgaris* and three lower plants. The scheme at the top depicts the locations and boundaries of the HR-A core, insert, and HR-B regions within the HR-A/B regions. The HR-A/B region of class B Hsfs is compact, whereas class A and class C Hsfs have extended HR-A/B regions caused by insertions of 21 (class A) or 7 (class C) amino acid residues between the A and B parts.
