# Supplementary material for: Genome Duplication and Gene Loss Affect the Evolution of Heat Shock Transcription Factor Genes in Legumes
Source: PLoS One. 2014 Jul 21;9(7):e102825. doi: 10.1371/journal.pone.0102825 (PMC4105503; doi:10.1371/journal.pone.0102825)
Supplement: Figure S3 — NJ phylogeny of A. thaliana , O. sativa , Z. mays , L. japonicus , M. truncatula , C. arietinum , G. max , C. cajan and P. vulgaris Hsf proteins. (PDF) [file pone.0102825.s003.pdf]

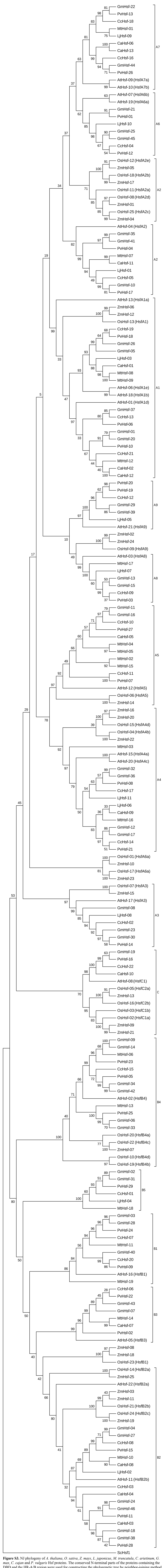

Figure S3. NJ phylogeny of *A. thaliana*, *O. sativa*, *Z. mays*, *L. japonicus*, *M. truncatula*, *C. arietinum*, *G. max*, *C. cajan* and *P. vulgaris* Hsf proteins. The conserved N-terminal parts of the proteins containing the DBD and the HR-A/B region were used for constructing the phylogenetic tree by neighbor-joining method. ScHsf1 was set as the outgroup. The legume Hsfs were grouped into three major classes, A, B and C. Class A and B were further divided into nine (A1–9) and five (B1–5) subgroups with high bootstrap values (>50).
