# Supplementary material for: Genome Duplication and Gene Loss Affect the Evolution of Heat Shock Transcription Factor Genes in Legumes
Source: PLoS One. 2014 Jul 21;9(7):e102825. doi: 10.1371/journal.pone.0102825 (PMC4105503; doi:10.1371/journal.pone.0102825)
Supplement: Figure S5 — The full syntenic maps of chromosome regions containing Hsf genes across L. japonicus , M. truncatula , G. max and C. cajan genes. (PDF) [file pone.0102825.s005.pdf]

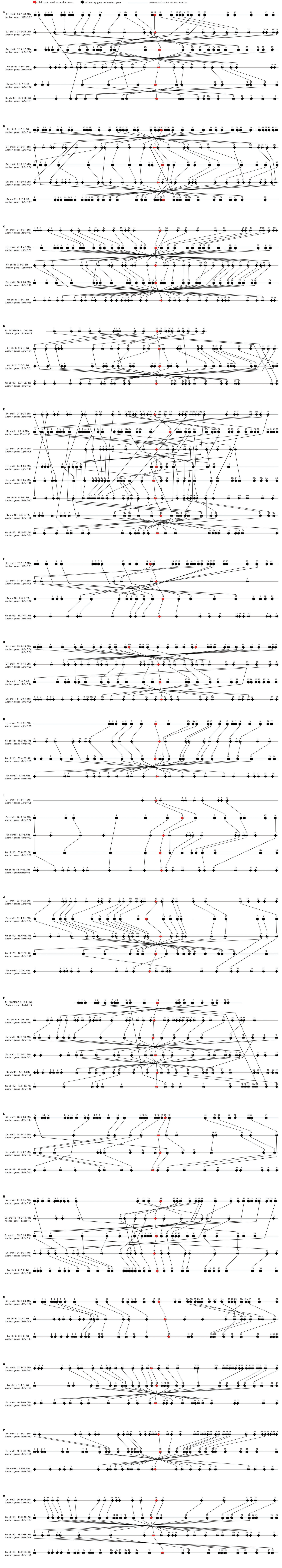

Figure S5. The full syntenic maps of chromosome regions containing Hsf genes across *L. japonicus*, *M. truncatula*, *G. max* and *C. cajan* genes. Figures in (A)–(Q) are 17 groups of syntenic segments. Arrows represent individual genes and their transcriptional orientations. Distances are roughly to scale. The Hsf genes are highlighted in red, and their flanking genes are shown in black. All genes are numbered from the left to the right for each interval. Duplicated genes are given the same number but ordered by a, b, c, …. Genes with syntenic matches are linked with lines.
