# Supplementary material for: Genome Duplication and Gene Loss Affect the Evolution of Heat Shock Transcription Factor Genes in Legumes
Source: PLoS One. 2014 Jul 21;9(7):e102825. doi: 10.1371/journal.pone.0102825 (PMC4105503; doi:10.1371/journal.pone.0102825)
Supplement: Figure S6 — Sliding window analysis employed to estimate selective pressures on 35 pairs of duplicated Hsf genes in G. max . (PDF) [file pone.0102825.s006.pdf]

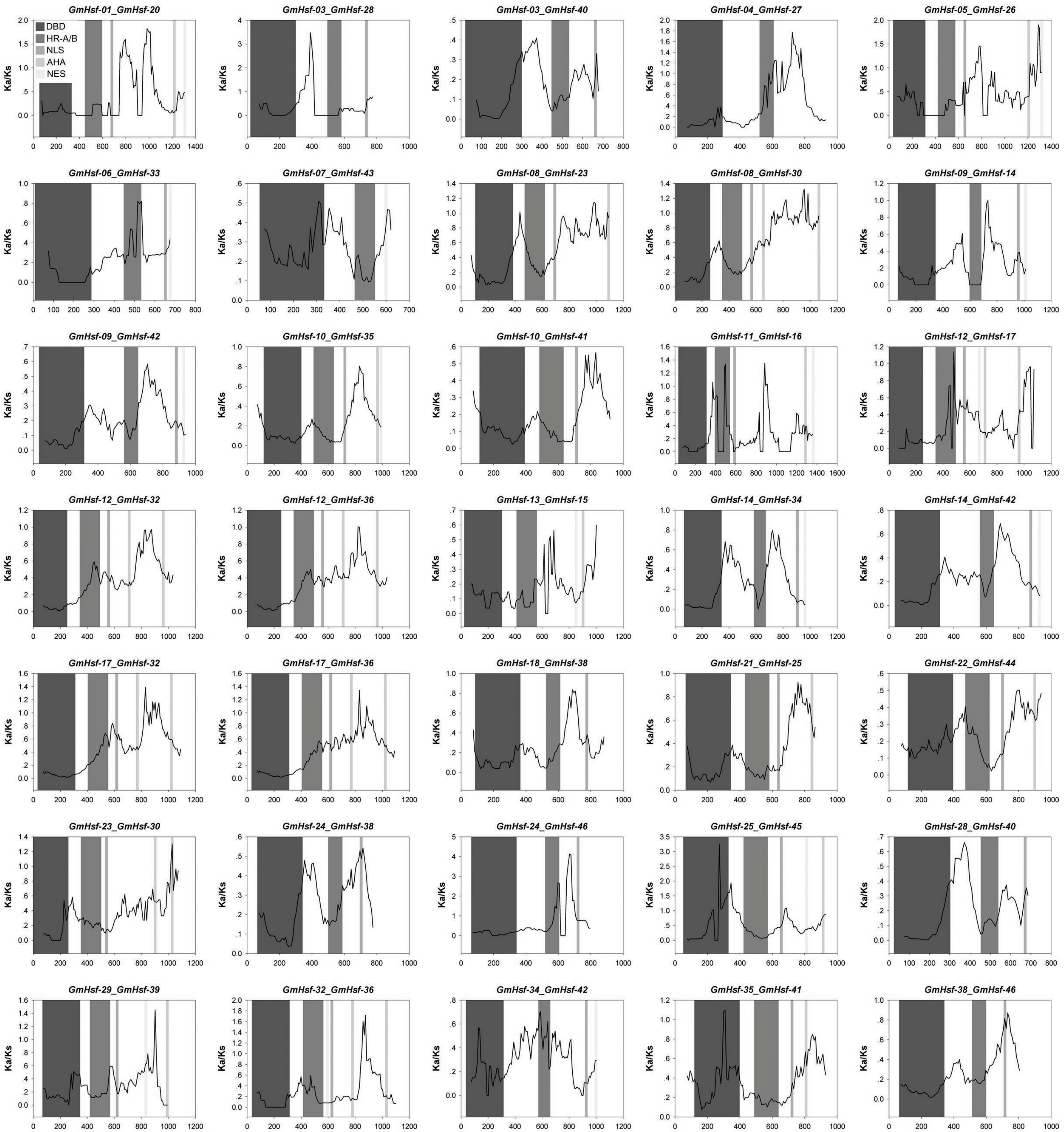

Figure S6. Sliding window analysis employed to estimate selective pressures on 35 pairs of duplicated Hsf genes in *G. max*. For sliding window analysis, nucleotide sequences of *G. max* Hsf genes are used. Window size is 150 bp, and step size is 9 bp. The gray blocks, from dark to light, indicate the positions of the DBD domain, HR-A/B region, NLS, NES and AHA motifs, respectively.
