# Supplementary material for: Genome Duplication and Gene Loss Affect the Evolution of Heat Shock Transcription Factor Genes in Legumes
Source: PLoS One. 2014 Jul 21;9(7):e102825. doi: 10.1371/journal.pone.0102825 (PMC4105503; doi:10.1371/journal.pone.0102825)
Supplement: Table S1 — Primers used in quantitative real-time PCR. (PDF) [file pone.0102825.s007.pdf]

Table S1. Primers used in quantitative real-time PCR

Gene name	Forword primers (5'-3')	Reverse primers (5'-3')
LjHsf-01	GTGGGTCCTCCTCCATTTCTC	GAGTCCCAGACGACGAAGCT
LjHsf-02	CTCCTCGCAGGAACCTGATG	TCCAACCAAGGCTCCTGATC
LjHsf-03	CCTGCTGTGTTGACGGATTG	TCTGTCCCCGATCCTTGAAC
LjHsf-04	AAGAAGTGCGAGCCAAGTGTGT	GCGTCTGCAGCTCCAATCTC
LjHsf-05	CTCTCCGACGACGGTTTCAG	TCGTGCAACCCTTCCATTG
LjHsf-06	GCCGGTTCACAGCCATTCTA	TGCCACTCGTGCTGATGTCT
LjHsf-07	CGACATGGTGGAGGACGATT	AAAGCGGTGATGTCGGAGAT
LjHsf-08	AGCGAGGGAAGAAGCAGTTG	TTCCCGGCATCAGAAGAAGA
LjHsf-09	CCCAGCCAATGGAAGGACTT	CATGGTTGGTAGCTGGATCGT
LjHsf-10	TCCTATTCTGGTGGCGTTCCT	CTGTTATTCCCTGTGCTCCATGA
LjHsf-11	AGTCACCCAGCATCGACATG	GGGCTGCTACTGCAGGTTGT
β-tubulin	CCCATTCCCTCGTCTCCATT	GATCAGCGGCACACATCATG
