# Supplementary material for: Genome Duplication and Gene Loss Affect the Evolution of Heat Shock Transcription Factor Genes in Legumes
Source: PLoS One. 2014 Jul 21;9(7):e102825. doi: 10.1371/journal.pone.0102825 (PMC4105503; doi:10.1371/journal.pone.0102825)
Supplement: Table S2 — Information about Hsfs in L. japonicus , M. truncatula , C. arietinum , G. max , C. cajan , P. vulgaris , S. moellendorffii , P. patens and C. reinhardtii . (PDF) [file pone.0102825.s008.pdf]

Table S2. Information about Hsfs in *L. japonicus*, *M. truncatula*, *C. arietinum*, *G. max*, *C. cajan*, *P. vulgaris*, *S. moellendorffii*, *P. patens* and *C. reinhardtii*.

Species	Gene name	Gene identifer	ORF (aa)	MW (Da)	pI	Genomic position
L. japonicus	*LjHsf-01*	chr1.CM0133.500.r2.m	387	43259.36	5.01	LjChr1:23590373..23591777
	LjHsf-02	chr2.CM0021.3250.r2.m	336	36577.72	5.1	LjChr2:31394445..31395549
	LjHsf-03	chr3.CM0127.360.r2.m	487	54185.41	5.04	LjChr3:46840532..46842190
	LjHsf-04	chr4.CM0432.1390.r2.a	208	24212.45	7.62	LjChr4:6965150..6966843
	LjHsf-05	chr4.CM0297.80.r2.a	392	44390.32	5.82	LjChr4:31165488..31167003
	LjHsf-06	chr4.CM0046.1600.r2.m	401	45706.92	5.34	LjChr4:36402041..36403354
	LjHsf-07	chr4.CM0042.1450.r2.m	367	41932.29	5.14	LjChr4:42520512..42522368
	LjHsf-08	chr5.CM0359.80.r2.m	512	57909.21	5.32	LjChr5:11584169..11586589
	LjHsf-09	chr5.CM0024.490.r2.m	331	38078.98	5.81	LjChr5:17733842..17735506
	LjHsf-10	chr5.CM0200.740.r2.m	354	41006.94	5.42	LjChr5:32187112..32188847
	LjHsf-11	chr6.CM0118.900.r2.d	395	44937.97	5.31	LjChr6:24525770..24527094
M. truncatula	*MtHsf-01*	Medtr1g071360.1	372	43603.44	5.68	MtChr1:17592134..17594193
	MtHsf-02	Medtr1g107080.1	329	37656.99	4.95	MtChr1:31581884..31583623
	MtHsf-03	Medtr2g017890.1	474	53638.98	5.24	MtChr2:5427722..5430795
	MtHsf-04	Medtr2g100670.1	419	47285.64	4.66	MtChr2:32133687..32137145
	MtHsf-05	Medtr2g100680.1	444	49984.17	6.55	MtChr2:32138306..32140457
	MtHsf-06	Medtr3g101870.1	373	42189.14	7.73	MtChr3:35982638..35985479
	MtHsf-07	Medtr3g104550.1	378	42625.82	4.94	MtChr3:36702717..36704919
	MtHsf-08	Medtr4g077970.1	493	54955.32	5.15	MtChr4:25511197..25514751
	MtHsf-09	Medtr4g078070.1	493	54955.32	5.15	MtChr4:25567635..25571189
	MtHsf-10	Medtr5g010680.1	359	39641.46	5.16	MtChr5:2665834..2669177
	MtHsf-11	Medtr5g017470.1	432	49356.85	8.86	MtChr5:6122009..6130936
	MtHsf-12	Medtr5g029680.1	502	55357.62	4.72	MtChr5:12227106..12231942
	MtHsf-13	Medtr5g089170.1	254	29617.47	6.45	MtChr5:37712245..37713757
	MtHsf-14	Medtr7g091370.1	233	26980.61	8.9	MtCh7:28797928..28800318
	MtHsf-15	Medtr8g083100.1	487	54344.07	5.05	MtChr8:22854701..22858145
	MtHsf-16	Medtr8g087540.1	401	45835.01	5.23	MtChr8:24376345..24378486
	MtHsf-17	Medtr8g105780.1	371	42183.31	5.07	MtChr8:31473372..31476090
	MtHsf-18	AC233659_12.1	210	24509.92	6.15	AC233659:14179947..14181646
	MtHsf-19	CU571152_1015.1	256	28580.25	6.37	CU571152.5:66438..76780
C. arietinum	*CaHsf-01*	TC01236	494	55088.64	5.06	n.d.
	CaHsf-02	TC02426	497	54812.79	4.79	n.d.
	CaHsf-03	TC04763	332	36929.52	6.85	n.d.
	CaHsf-04	TC04961	267	30044.06	8.39	n.d.
	CaHsf-05	TC05890	482	54488.23	5.24	n.d.
	CaHsf-06	TC07370	351	40615.67	5.19	n.d.
	CaHsf-07	TC09172	240	27627.17	8.52	n.d.
	CaHsf-08	TC10527	337	37124.35	5.43	n.d.
	CaHsf-09	TC11068	401	45693.15	5.28	n.d.
	CaHsf-10	TC12461	258	29873.94	5.32	n.d.
	CaHsf-11	TC15059	367	41782.48	4.83	n.d.
	CaHsf-12	TC34529	473	52722.63	4.87	n.d.
	CaHsf-13	TC34660	310	35746.43	6.64	n.d.
G. max	*GmHsf-01*	Glyma01g01990.1	461	50843.64	5.31	GmChr1:1541377..1544307
	GmHsf-02	Glyma01g34490.1	209	24228.42	7.01	GmChr1:46835978..46838383
	GmHsf-03	Glyma01g39260.1	282	31193.84	9.01	GmChr1:51227519..51229606
	GmHsf-04	Glyma01g42640.1	338	36922.86	5.14	GmChr1:53862802..53863906
	GmHsf-05	Glyma01g44330.1	464	51846.82	4.93	GmChr1:55025297..55027987
	GmHsf-06	Glyma02g44670.1	291	34062.94	6.59	GmChr2:49172352..49173651
	GmHsf-07	Glyma03g29190.1	231	26714.21	8.8	GmChr3:37141517..37145012
	GmHsf-08	Glyma03g34900.1	423	48268.08	5.58	GmChr3:42204941..42207241
	GmHsf-09	Glyma04g04200.1	363	40646.71	8.41	GmChr4:3095592..3096844
	GmHsf-10	Glyma04g05500.2	372	41922.78	5.08	GmChr4:4167574..4169572
	GmHsf-11	Glyma05g28460.1	479	54028.91	5.43	GmChr5:34265160..34270029
	GmHsf-12	Glyma05g29470.1	382	43805.67	5.01	GmChr5:35079785..35081242
	GmHsf-13	Glyma05g34450.1	358	41018.33	5.23	GmChr5:38783656..38785937
	GmHsf-14	Glyma06g04390.1	363	40469.37	8.44	GmChr6:3057619..3058862
	GmHsf-15	Glyma08g05220.1	364	41717.06	5.14	GmChr8:3703299..3706068
	GmHsf-16	Glyma08g11460.1	477	53896.74	5.66	GmChr8:8333717..8338512
	GmHsf-17	Glyma08g12630.1	402	45953.05	5.07	GmChr8:9242535..9244700
	GmHsf-18	Glyma09g26510.1	324	35584.71	5.73	GmChr9:32991190..32992574
	GmHsf-19	Glyma09g32300.1	320	35975.78	6.28	GmChr9:38833147..38834811
	GmHsf-20	Glyma09g33920.1	500	55532.95	4.91	GmChr9:40400230..40403665
	GmHsf-21	Glyma10g00560.1	324	37698.14	4.63	GmChr10:320843..322652
	GmHsf-22	Glyma10g03530.1	341	39741.93	5.69	GmChr10:2568451..2569878
	GmHsf-23	Glyma10g07620.1	435	48910.19	5.72	GmChr10:6371342..6374987
	GmHsf-24	Glyma10g38240.1	289	32546.75	7.05	GmChr10:46052164..46053421
	GmHsf-25	Glyma10g38930.1	448	52058.88	5.96	GmChr10:46662541..46665008
	GmHsf-26	Glyma11g01190.1	464	52020.26	5.33	GmChr11:684016..687478
	GmHsf-27	Glyma11g02800.1	355	38652.8	4.96	GmChr11:1807814..1810003
	GmHsf-28	Glyma11g06010.1	285	31585.34	9.11	GmChr11:4245812..4247876
	GmHsf-29	Glyma13g16510.1	368	42400.06	6.98	GmChr13:20463262..20465356
	GmHsf-30	Glyma13g21490.1	428	48234.94	5.15	GmChr13:25054276..25057239
	GmHsf-31	Glyma13g24860.1	213	24798.1	7.64	GmChr13:28171233..28173625
	GmHsf-32	Glyma13g29760.1	392	44933.77	4.89	GmChr13:32612241..32614169
	GmHsf-33	Glyma14g04070.1	250	29124.02	8.87	GmChr14:2700334..2701445
	GmHsf-34	Glyma14g09190.1	370	42035.88	7.72	GmChr14:7208889..7210925
	GmHsf-35	Glyma14g11030.1	362	41051.65	4.78	GmChr14:9314476..9317368
	GmHsf-36	Glyma15g09280.1	392	44878.89	5.08	GmChr15:6616217..6618219
	GmHsf-37	Glyma16g13400.1	510	56348.77	4.92	GmChr16:14375544..14380501
	GmHsf-38	Glyma16g32070.1	348	37759	6.12	GmChr16:35256650..35258245
	GmHsf-39	Glyma17g06160.1	360	41608.04	8.53	GmChr17:4358386..4360467
	GmHsf-40	Glyma17g20070.1	282	30982.62	6.03	GmChr17:18609225..18613267
	GmHsf-41	Glyma17g34540.1	336	38138.23	5.17	GmChr17:38530630..38533545
	GmHsf-42	Glyma17g35980.1	364	41570.35	8.47	GmChr17:39960997..39962650
	GmHsf-43	Glyma19g31940.1	233	26787.39	9.39	GmChr19:39720668..39723322
	GmHsf-44	Glyma19g34210.1	370	42387.41	5.44	GmChr19:41827148..41829192
	GmHsf-45	Glyma20g28870.1	341	39451.63	5.16	GmChr20:37820334..37821899
	GmHsf-46	Glyma20g29610.1	300	33295.55	8.73	GmChr20:38484532..38485826
C. cajan	*CcHsf-01*	C.cajan_19915	205	23661	7.58	CcChr1:7615927..7617782
	CcHsf-02	C.cajan_06276	410	46558.38	5.51	CcChr2:18828876..18832166
	CcHsf-03	C.cajan_07385	251	28297.08	9.24	CcChr2:30447647..30448481
	CcHsf-04	C.cajan_07476	328	38364.1	5.43	CcChr2:31450060..31451723
	CcHsf-05	C.cajan_09277	337	38286.89	5.39	CcChr3:12799267..12800550
	CcHsf-06	C.cajan_09456	231	26740.16	9.08	CcChr3:14507414..14510213
	CcHsf-07	C.cajan_12769	289	32419.51	9.29	CcChr6:18256391..18257755
	CcHsf-08	C.cajan_13182	352	38389.47	5.21	CcChr6:22270411..22271556
	CcHsf-09	C.cajan_15551	362	41652.91	5	CcChr8:2169380..2171458
	CcHsf-10	C.cajan_01041	404	45615.51	5.86	CcChr11:11017250..11021000
	CcHsf-11	C.cajan_03188	443	49847.83	5.02	CcChr11:35100306..35101777
	CcHsf-12	C.cajan_03745	360	41267.43	5.4	CcChr11:41298162..41299801
	CcHsf-13	C.cajan_25281	514	56492.73	4.81	CcScaffold000155:676508..683343
	CcHsf-14	C.cajan_32266	353	40801.49	5.41	CcScaffold000182:110140..111471
	CcHsf-15	C.cajan_33410	304	34943.21	8.71	CcScaffold000208:172995..174383
	CcHsf-16	C.cajan_30804	342	39883.92	5.8	CcScaffold000221:157822..159747
	CcHsf-17	C.cajan_29530	347	40099.85	5.42	CcScaffold128446:59890..61178
	CcHsf-18	C.cajan_30373	342	39615.96	5.72	CcScaffold128680:155900..157182
	CcHsf-19	C.cajan_24088	449	50349.2	5.19	CcScaffold130941:699987..702801
	CcHsf-20	C.cajan_37173	297	32824.83	6.96	CcScaffold132487:91136..94149
	CcHsf-21	C.cajan_36375	493	54295.56	4.79	CcScaffold132641:25714..29499
	CcHsf-22	C.cajan_27606	303	34299.81	5.82	CcScaffold132776:531150..532716
P. vulgaris	*PvHsf-01*	Phvulv091021617m	334	38667.39	4.82	PvScaffold00001:251683..253586
	PvHsf-02	Phvulv091021516m	241	28258.45	9.2	PvScaffold00001:2697465..2700690
	PvHsf-03	Phvulv091020489m	360	41209.45	4.9	PvScaffold00002:4048025..4051185
	PvHsf-04	Phvulv091010948m	373	42462.58	4.91	PvScaffold00004:136234..138819
	PvHsf-05	Phvulv091010839m	339	38843.55	7.31	PvScaffold00004:1726980..1728693
	PvHsf-06	Phvulv091011519m	474	52777.74	4.9	PvScaffold00005:2620246..2624309
	PvHsf-07	Phvulv091015583m	441	49829.8	5.61	PvScaffold00008:147554..149599
	PvHsf-08	Phvulv091022066m	386	43876.73	5.01	PvScaffold00013:1987221..1989485
	PvHsf-09	Phvulv091016481m	278	30720.38	5.92	PvScaffold00017:1210184..1213352
	PvHsf-10	Phvulv091016314m	490	54230.43	5.02	PvScaffold00022:22643..26535
	PvHsf-11	Phvulv091023300m	304	34047.3	7.02	PvScaffold00029:513050..514633
	PvHsf-12	Phvulv091023279m	353	40995.34	5.29	PvScaffold00029:1336185..1338209
	PvHsf-13	Phvulv091029236m	357	41497.85	5.33	PvScaffold00036:796060..798714
	PvHsf-14	Phvulv091005584m	452	51271.3	4.83	PvScaffold00037:1087962..1091067
	PvHsf-15	Phvulv091008650m	356	38917.31	4.93	PvScaffold00056:802951..804097
	PvHsf-16	Phvulv091014509m	400	44917.19	6.9	PvScaffold00072:257107..258930
	PvHsf-17	Phvulv091000949m	373	41688.35	4.9	PvScaffold00082:292167..294440
	PvHsf-18	Phvulv091027740m	465	51726.58	5.26	PvScaffold00115:203936..207546
	PvHsf-19	Phvulv091027312m	404	46423.45	5.83	PvScaffold00117:348208..350665
	PvHsf-20	Phvulv091027304m	404	46308.2	5.96	PvScaffold00117:372246..374560
	PvHsf-21	Phvulv091025487m	403	45804.06	5.14	PvScaffold00123:218774..221028
	PvHsf-22	Phvulv091031418m	232	26585.97	9.08	PvScaffold00145:476409..478902
	PvHsf-23	Phvulv091019741m	348	39112.16	8.85	PvScaffold00167:117106..118295
	PvHsf-24	Phvulv091019125m	284	31522.5	8.44	PvScaffold00174:14267..16334
	PvHsf-25	Phvulv091002558m	269	31202.45	7.22	PvScaffold00211:450675..452077
	PvHsf-26	Phvulv091018584m	366	42242.3	5.54	PvScaffold00228:76161..79130
	PvHsf-27	Phvulv091013313m	487	54538.32	5.44	PvScaffold00257:84991..89864
	PvHsf-28	Phvulv091027109m	340	37155.59	5.62	PvScaffold00456:114542..116347
	PvHsf-29	Phvulv091001383m	206	23907.29	7.61	PvScaffold00794:30956..32738
S. moellendorffii	*SmHsf-01*	92634	319	35206.53	6.83	SmScaffold_13:929766..931086
P. patens	*PpHsf-01*	Pp1s12_41V6.1	404	45295.84	6.65	PpScaffold_12:263670..266592
	PpHsf-02	Pp1s19_153V6.1	417	47038.29	8.13	PpScaffold_19:1060316..1063077
	PpHsf-03	Pp1s42_157V6.1	467	52526.2	5.19	PpScaffold_42:1347549..1352410
	PpHsf-04	Pp1s84_95V6.1	525	57650.05	4.93	PpScaffold_84:563949..567070
	PpHsf-05	Pp1s85_27V6.1	413	46035.97	7.63	PpScaffold_85:161245..163446
	PpHsf-06	Pp1s207_20V6.1	455	49222.33	7.66	PpScaffold_207:163634..165778
	PpHsf-07	Pp1s249_84V6.1	529	58119.41	4.77	PpScaffold_249:545270..549261
C. reinhardtii	*CrHsf-01*	Cre07.g354500.t1.2	1248	117501.12	5.98	CrChr7:5699452..5706998
	CrHsf-02	Cre09.g387150.t1.1	801	78393.22	5.44	CrChr9:247570..254161

MW, Molecular weight in Dalton; pI, Isoelectric point.

n.d., not determined.
