# Supplementary material for: Genome Duplication and Gene Loss Affect the Evolution of Heat Shock Transcription Factor Genes in Legumes
Source: PLoS One. 2014 Jul 21;9(7):e102825. doi: 10.1371/journal.pone.0102825 (PMC4105503; doi:10.1371/journal.pone.0102825)
Supplement: Table S3 — Domain and motif survey of legume and three lower plant Hsfs. (PDF) [file pone.0102825.s009.pdf]

Table S3. Domain and motif survey of legume and three lower plants Hsfs.

Gene name	Group	DBD	OD	NLS	NES	AHA1	AHA2
LjHsf-03	A1	2-95	132-182	(206) KKRR		(424) DEFWEQFF	
MtHsf-08	A1	10-103	140-190	(214) KKRR	(484) LLASDSTL	(424) DEFWEQFFRP	
MtHsf-09	A1	10-103	140-190	(214) KKRR	(484) LLASDSTL	(424) DEFWEQFFRP	
MtHsf-12	A1	30-123	161-211	(236) KKRR	(424) LPIDLDCL	(443) SSIWDDLL	
CaHsf-01	A1	10-103	140-190	(214) KKRR	(479) LTQQMELL	(425) DEFWELFFM	
CaHsf-02	A1	30-120	158-208	(233) KKRR	(480) LDQLTEQM	(439) PSIWDDLLQT	
CaHsf-12	A1	3-96	134-184	(209) KKRR	(465) LLSSDR	(415) PSIWDDLLQT	
GmHsf-01	A1	18-111	149-199	(224) KKRR	(453) LLSSD	(403) NPIWDEIL	
GmHsf-05	A1	12-105	141-191	(216) KKRR	(453) LTQQMGL	(399) DEFWELFLRP	
GmHsf-20	A1	18-121	159-209	(234) KKRR	(492) LLSSN	(442) NPIWDEIL	
GmHsf-26	A1	12-105	141-191	(216) KKRR	(453) LTQQMGL	(399) DEFWELLLRP	
GmHsf-37	A1	28-121	158-208	(233) KKRR	(502) LLSSD	(452) PHFWDDILRT	
CcHsf-13	A1	31-124	162-212	(236) KKRR	(506) LLSSDT	(456) PHFWDDIVRT	
CcHsf-19	A1	1-84	120-170	(195) KKRR	(438) LTQQMGLL	(384) DEFWELFFRP	
CcHsf-21	A1	18-111	148-198	(224) KKRR	(485) LLSSD	(435) PSIWDDLLQT	
PvHsf-06	A1	25-118	156-206	(231) KKRR	(466) LLSSD	(416) PNFWDDIV	
PvHsf-10	A1	18-111	149-199	(224) KKRR	(482) LLSSDT	(432) PSIWDEILQT	
PvHsf-18	A1	12-105	141-191	(216) KKRR	(454) LTKQMGLL	(400) DEFWELFFRP	
PpHsf-03	A1	43-136	163-213	(234) RKKRR		(405) SGFWEQFLTE	
PpHsf-04	A1	56-149	190-240	(263) RKKRR	(467) LDGDLDL	(449) DIFWEQFL	
PpHsf-07	A1	56-149	192-242	(265) RKKRR	(472) LDHDLDL	(454) DIFWEQFLSE	
LjHsf-01	A2	46-139	169-219	(244) RRKRR	(376) LVDHMGYL	(291) GSIFSAAYDN	(367) DDWTEDLQD
MtHsf-07	A2	44-137	168-218	(239) KRKRR	(244) LTASLSLENL	(321) DAVWEDLL	(361) DNWTVDLQK
CaHsf-11	A2	45-138	170-220	(241) KRKRR	(359) LVDQMDF	(309) DTVWEDLLNQ	(349) DDDWTVDLQN
GmHsf-10	A2	41-134	166-216	(239) RRKRR	(361) LVDHMGYL	(312) DSVWEDLLNQ	(351) ADEWSEDLQN
GmHsf-35	A2	41-134	164-214	(239) NRKRR	(354) LVDHMDYL	(280) DTFFSPAYDN	(345) DDWSEQLQD
GmHsf-41	A2	40-133	163-213	(239) NRKRR		(268) DTFFAPAYDN	
CcHsf-05	A2	40-133	163-213	(236) RRKRR	(326) LVDHMGYL	(269) ESFFSAACDD	(316) DDEWNEDLQN
PvHsf-04	A2	43-135	164-214	(240) NRKRR	(345) LEDLVAN	(280) DTFFSPAFDS	(353) DDWMEQFQD
PvHsf-17	A2	43-136	166-216	(239) RRKRR	(362) LVDHLGHL	(313) DAIWEDLLNQ	(353) DEWSEDLQN
LjHsf-08	A3	92-185	214-264	(276) KKK8RVRRK		(311) PDWGNIIM	
GmHsf-08	A3	37-130	159-209	(230) PKVRRRF		(259) QPDWRNV	(412) DMWDINYL
GmHsf-23	A3	45-138	169-219	(229) RHKK8RVVRK		(351) EDIWDSGLNV	(393) SDIWDIGLG
GmHsf-30	A3	1-87	118-168	(189) RVVRK	(393) LGSLGID	(218) HDWRNIGM	(344) EDIWDSGLNV
CcHsf-02	A3	17-110	141-191	(201) RHKK	(312) LDFSALGI	(242) PDWRIIGMS	(326) EDIWDCDLNV
PvHsf-14	A3	20-113	144-194	(204) RHKK		(245) PDWRNIDMSS	(368) EDIWDSDLNL
LjHsf-06	A4	11-104	135-185	(203) RKRR	(240) LTLNMERLDQ	(254) MLFWETIAHE	(337) DVFWEQFLTE
LjHsf-11	A4	11-104	136-186	(204) RKRR	(248) LDSLES	(255) MTFWENIVHD	(340) DVFWEQFLTE
MtHsf-03	A4	12-105	139-189	(194) KKEQKRK	(234) LSLNLEQLDLL	(248) MTFWEEITND	(353) NEFWGQYL
MtHsf-16	A4	10-103	134-184	(202) RKRR	(240) LTLNTERL	(254) VAFWETLA	(337) DVFWEQFLTE
CaHsf-09	A4	11-104	135-185	(204) RKRR	(243) LNAERLDQL	(255) MVFWETIAHE	(337) DVFWEQFLTE
GmHsf-12	A4	1-84	115-165	(183) RKRR	(220) LTLNVERLDQL	(234) MAFWEAIVHD	(318) DVFWEQFLTE
GmHsf-17	A4	11-104	135-185	(203) RKRR	(240) LTLNVERLDQL	(254) MAFWEAIVHD	(338) DVFWEQFLTE
GmHsf-32	A4	12-105	138-188	(206) RKRR	(195) LNLLLL	(257) MTFWENITHD	(341) DIFWERFLTE
GmHsf-36	A4	12-105	138-188	(206) RKRR	(195) LNLLLL	(257) MTFWEDITHD	(341) DIFWERFLTE
CcHsf-14	A4	11-104	135-185	(203) RKRR	(240) LTLNMERLDQL	(254) MAFWEAIAHD	(289) DVFWEQFLTE
CcHsf-17	A4	11-104	137-187	(206) RKRR	(252) LLESSI	(257) IEFWEEMSHD	(299) DIFWEKFLTE
PvHsf-08	A4	12-105	138-188	(206) RKRR		(257) IMFWENIAHD	(336) DIFWERFLTE
PvHsf-21	A4	12-105	136-186	(204) RKRR	(241) LALNVERLDQL	(255) VAFWEAIAHD	(339) DVFWEQFLTE
MtHsf-02	A5	20-113	140-189	(200) RK10KKRRL	(257) LSLADLDM	(307) DVFWEQFLTE	
MtHsf-04	A5	139-232	259-309	(317) HVRR	(356) LRLELS		
MtHsf-05	A5	54-147	174-224	(234) KRK10KKRR	(349) ISRQLNLTL	(435) DLFWEQFLTE	
MtHsf-15	A5	20-113	140-189	(200) RK10KKRRL	(253) LRLELS	(435) DVFWENFLTE	
CaHsf-05	A5	15-108	135-185	(208) KKRR	(477) MDNLTL	(431) DVFWEQFLTE	
GmHsf-11	A5	13-106	133-183	(194) RK9KKRRLP	(474) MDQLTL	(427) DVFWEQFLTE	
GmHsf-16	A5	11-104	131-181	(192) RK9KKRRLP	(472) MDQLTL	(425) DVFWEQFLTE	
CcHsf-10	A5	13-106	133-183	(194) RK10KKRR	(399) MDQLTL	(352) DVFWEQFLTE	
CcHsf-11	A5	14-107	131-181	(192) RR10KKRR	(438) MDQLTL	(391) DVFWEQFLTE	
PvHsf-07	A5	12-105	131-181	(204) KKRR		(389) DVFWEQFLTE	
PvHsf-27	A5	18-111	138-188	(211) KKRR	(482) MDQLRL	(435) DVFWEQFLTE	
LjHsf-10	A6	38-131	160-210	(223) KEWRK7KKRRR	(338) LAEQLGYL	(313) EVFWQDLLDE	
GmHsf-21	A6	22-115	145-195	(211) RK10KRIRS	(312) LAEELGYL	(283) EVLWEELLNE	
GmHsf-25	A6	124-217	251-301	(326) NKKRR	(380) LDLALNL	(415) EVFWQDLLNE	
GmHsf-45	A6	17-110	142-192	(218) KKRRR	(270) LPLVLNL	(304) EVFWQDLLNE	
CcHsf-04	A6	38-131	162-212	(237) KKRRR	(315) LAEQLGYL	(293) EVFWQDLLNE	
PvHsf-01	A6	34-127	153-203	(229) KRRR	(323) LSQELGYL	(303) EGVEEDLLAL	
PvHsf-12	A6	42-135	165-215	(240) KKRR	(287) LDLGLNL	(319) EVFWQDLLKE	
LjHsf-09	A7	32-125	150-200	(213) KEKRK7RKRR		(304) EEFWEALLFS	
MtHsf-01	A7	27-120	140-190	(205) KKK9KKRRL	(253) LEMLALEM	(292) EGFWEDLMFS	
CaHsf-06	A7	38-131	162-212	(225) KEKRK7KKRRR		(309) EGFGEELFSE	
CaHsf-13	A7	38-131	162-212	(225) KEKRK7KKRRR	(273) LEVLAMEM		
GmHsf-22	A7	39-132	158-208	(232) KKRR	(330) LANQLDLL	(298) EEFWEELLFS	
GmHsf-44	A7	46-139	173-223	(236) KEKRK7KKRRR	(292) LEVLAM	(328) EGFWEELFSE	
CcHsf-16	A7	48-141	171-221	(234) KEKRK7KKRRR	(265) LELLAMEM	(301) EGFWEEFFSE	
CcHsf-18	A7	35-128	154-204	(228) KKRR	(331) LANQLGCF	(299) EEFWEELLCS	
PvHsf-13	A7	41-134	160-210	(234) KKRR	(346) LANELGCL	(314) EEFWEELLIS	
PvHsf-26	A7	46-139	168-218	(231) KEKRK7KKRRR	(286) LEVLALEM	(322) EGFWEELFSE	
LjHsf-07	A8	14-108	144-194	(246) KRKH	(272) LKDLCISSEFL	(307) DGSWEQLFLG	
MtHsf-17	A8	20-114	151-201	(253) KLKPVVPR	(287) LKLLLDEKLCPL	(312) DGSWEQLFLG	
GmHsf-13	A8	8-101	137-187		(281) LSPLEN	(298) DGSWEQLFLG	
GmHsf-15	A8	14-107	143-193		(287) LSPLEN	(304) DGSWEQLFLG	
CcHsf-09	A8	12-105	141-191		(285) LSPLEN	(302) DGLWEQLFLG	
PvHsf-03	A8	12-105	141-191		(265) LKDLCI	(300) DGSWEQLFLG	
LjHsf-05	A9	60-153	179-229	(243) KRKR8KRPRL	(361) LELEDL		
GmHsf-29	A9	32-125	149-199	(213) RRKR8KRPRL	(287) LEDELCSSLQGL	(353) TDWSVGSASG	
GmHsf-39	A9	27-120	144-194	(208) RRKR8KRPRL	(279) LEDELGNSLQGL	(345) TDWSVGSASG	
CcHsf-12	A9	59-152	176-226	(240) RRKR8KRPRL	(334) IYLELEDL	(346) TDWVGSAGGL	
PvHsf-19	A9	70-163	187-237	(251) RRKR8KRPRL	(379) LELEDL	(389) TDWSLGSTFG	
PvHsf-20	A9	70-163	187-237	(251) RRKR8KRPRL	(379) LELEDL	(389) TDWSLGSAFG	
CrHsf-02	A	8-101	161-211	(248) RRKRR	(742) LSGLDL		
MtHsf-11	B1	114-207	271-300	(355) KKR	(406) LHPLEREILTL		
MtHsf-19	B1	7-100	145-174	(238) KRGR	(152) LTVELTL		
GmHsf-03	B1	7-100	165-194	(243) NHKRGR			
GmHsf-28	B1	8-101	167-196	(246) NHKRGR			
GmHsf-40	B1	9-102	153-182	(262) RKRGR			
CcHsf-07	B1	7-123	183-212	(262) KRGR			
CcHsf-20	B1	7-123	176-205	(278) RKR			
PvHsf-09	B1	7-100	152-181	(259) RKR			
PvHsf-24	B1	7-100	166-195	(247) KRVR	(270) FEPLNL		
LjHsf-02	B2	20-113	187-216	(280) KRHR	(240) LELIPAKDL		
MtHsf-10	B2	22-115	190-219	(300) KRCR	(246) LDLLPLRNVSL		
CaHsf-03	B2	27-120	191-220	(276) KRTR			
CaHsf-04	B2	21-114	164-193	(241) KRAR			
CaHsf-08	B2	22-115	187-216	(280) KRFR			
GmHsf-04	B2	5-98	174-203				
GmHsf-18	B2	31-124	179-208	(262) KRAR			
GmHsf-24	B2	21-114	173-202	(238) KRAR			
GmHsf-27	B2	22-115	192-221				
GmHsf-38	B2	30-123	180-209	(279) KRAR			
GmHsf-46	B2	21-114	178-207	(242) KKRAR			
CcHsf-03	B2	19-112	143-172	(212) KRGR			
CcHsf-08	B2	22-115	190-219	(286) KRCR			
PvHsf-11	B2	21-114	177-206	(249) KRSR			
PvHsf-15	B2	22-115	190-219	(292) KRCR			
PvHsf-28	B2	28-121	182-211	(275) KRAR			
MtHsf-14	B3	20-113	153-182	(154) KK12KRK	(174) LLDLVSI		
CaHsf-07	B3	20-113	157-186	(172) KRKCK	(178) LLDLVA		
GmHsf-07	B3	18-111	155-184	(170) KRKCK	(198) LFGVRL		
GmHsf-43	B3	18-113	157-186	(172) KRKCK	(200) LFGVRL		
CcHsf-06	B3	22-115	155-184	(170) KRKCK	(196) PKLFGVRL		
PvHsf-02	B3	57-150	187-216	(202) KRKCR	(228) LSFRLRL		
PvHsf-22	B3	19-112	156-185	(171) KRKCK	(199) LFGVRLDV		
MtHsf-06	B4	22-115	201-230	(320) KTK8KKR	(363) LGLNLM		
MtHsf-13	B4	23-116	166-195	(245) KKRVH	(212) LKLVEL		
GmHsf-06	B4	23-116	169-198	(261) KKRLH	(268) LEELHMMIISL		
GmHsf-09	B4	22-115	199-228	(317) KTK8KKR	(348) LEKDDLGLNL		
GmHsf-14	B4	22-115	200-229	(317) KTK8KKR	(348) LEKDDLGLNL		
GmHsf-33	B4	3-96	149-178	(238) KKRLH	(193) LKLLELDS		
GmHsf-34	B4	22-115	201-230	(318) KTK8KKR	(354) LENDELLGLNL		
GmHsf-42	B4	12-105	194-223	(310) KTK8KKR	(348) LENDELLGLNL		
CcHsf-15	B4	22-115	149-178	(249) RCKTK8KKR	(292) LGLNLM		
PvHsf-05	B4	22-115	191-220	(286) KTK8KKR	(327) FGLNLM		
PvHsf-23	B4	22-115	181-210	(302) KTK8KKR	(338) LGLNLM		
PvHsf-25	B4	23-116	168-197	(259) KKR			
SmHsf-01	B4	22-101	192-221	(270) RKR	(292) IELELM		
PpHsf-01	B4	23-116	176-205				
PpHsf-02	B4	23-116	178-207				
PpHsf-05	B4	23-116	178-207	(350) KRR	(401) LLICA		
PpHsf-06	B4	26-119	181-210		(437) LGLEL		
LjHsf-04	B5	29-126	168-197	(203) KVRK	(173) LELQTQI		
MtHsf-18	B5	31-128	170-199	(205) KVRR	(175) LELQM		
GmHsf-02	B5	32-130	169-198	(204) KVRR	(174) LELQVQI		
GmHsf-31	B5	35-133	173-202	(208) KVRR	(178) LELQM		
CcHsf-01	B5	32-128	165-194	(200) KVRK	(170) LELQM		
PvHsf-29	B5	33-129	166-195	(201) KLRK	(171) LELQM		
CrHsf-01	B	14-107	654-683		(832) LEKQLL		
CaHsf-10	C	10-103	138-174	(195) KKKPR	(200) LILPE		
GmHsf-19	C	14-107	142-178	(199) KKRR			
CcHsf-22	C	17-110	138-174	(195) KRRR			
PvHsf-16	C	97-190	223-259	(280) KKRR			

Numbers indicate the position of putative domains and motifs in Hsf.

Putative NLS can be monopartite (e.g., LjHsf-03, GmHsf-05) or bipartite (e.g., MtHsf-01, CcHsf-16).
