# Supplementary material for: Genome Duplication and Gene Loss Affect the Evolution of Heat Shock Transcription Factor Genes in Legumes
Source: PLoS One. 2014 Jul 21;9(7):e102825. doi: 10.1371/journal.pone.0102825 (PMC4105503; doi:10.1371/journal.pone.0102825)
Supplement: Table S4 — Likelihood ratio tests and parameter estimations for the six site models based on the coding sequences of G. max Hsf genes. (PDF) [file pone.0102825.s010.pdf]

Table S4. Likelihood ratio tests and parameter estimations for the six site models based on the coding sequences of *G. max* Hsf genes.

Site Model	Assumption	Estimates of Parameters	-InL	LRT Statistic	Df	chi-square(0.01)	Positively selected sites
**Data set: *G. max* Hsf family**							
M0	One-ratio	ω=0.15765	11472.31973				None
M1a	Nearly neutral	p_0=0.64506, p_1=0.35494<br>ω_0=0.11679, ω_1=1	11214.72377				Not allowed
M2a	Selection	p_0=0.64506, p_1=0.16967, p_2=0.18527<br>ω_0=0.11679, ω_1=1, ω_2=1	11214.72377	M1a VS M2a: 0	2	9.21	None
M3	Discrete	p_0=0.23362, p_1=0.40160, p_2=0.36478<br>ω_0=0.00441, ω_1=0.10859, ω_2=0.39845	10974.62579	M0 VS M3: 995.38788	4	13.28	None
M7	β	p=0.53920,q=2.23895	10966.95744				Not allowed
M8	β & ω	p_0=0.99999, (p_1=0.00001), p=0.53920, q=2.23895<br>ω=5.47020	10966.95887	M7 VS M8: 0.00286	2	9.21	None
**Data set: Class A *G. max* Hsfs**							
M0	One-ratio	ω=0.20051	11029.63101				None
M1a	Nearly neutral	p_0=0.70765, p_1=0.29235<br>ω_0=0.14421, ω_1=1	10783.30678				Not allowed
M2a	Selection	p_0=0.70765, p_1=0.22183, p_2=0.07052<br>ω_0=0.14421, ω_1=1, ω_2=1	10783.30678	M1a VS M2a: 0	2	9.21	None
M3	Discrete	p_0=0.29258, p_1=0.46116, p_2=0.24626<br>ω_0=0.02343, ω_1=0.19043, ω_2=0.61686	10642.48282	M0 VS M3: 774.29638	4	13.28	None
M7	β	p=0.62699,q=1.88816	10639.24953				Not allowed
M8	β & ω	p_0=0.98516, (p_1=0.01484), p=0.64256, q=2.02031<br>ω=1	10639.19731	M7 VS M8: 0.10444	2	9.21	127 Q
**Data set: Class B *G. max* Hsfs**							
M0	One-ratio	ω=0.10951	4928.91152				None
M1a	Nearly neutral	p_0=0.79009, p_1=0.20991<br>ω_0=0.07064, ω_1=1	4813.05665				Not allowed
M2a	Selection	p_0=0.79009, p_1=0.15427, p_2=0.05564<br>ω_0=0.07064, ω_1=1, ω_2=1	4813.05665	M1a VS M2a: 0	2	9.21	None
M3	Discrete	p_0=0.35383, p_1=0.43807, p_2=0.20810<br>ω_0=0.00704, ω_1=0.09464, ω_2=0.44126	4738.37109	M0 VS M3: 381.08086	4	13.28	None
M7	β	p=0.44819,q=2.76153	4740.65890				Not allowed
M8	β & ω	p_0=0.99999, (p1=0.00001), p=0.44819, q=2.76153<br>ω=27.16606	4740.66047	M7 VS M8: 0.00314	2	9.21	None

Positively selected sites were inferred based on posterior probabilities calculated by the method of Bayes Empirical Bayes as only implemented under the models of M2a and M8.
